# n-3 PUFA biosynthesis by the copepod *Apocyclops royi* documented using fatty acid profile analysis and gene expression analysis

**DOI:** 10.1242/bio.038331

**Published:** 2019-02-05

**Authors:** Bolette Lykke Holm Nielsen, Louise Gøtterup, Tue Sparholt Jørgensen, Benni Winding Hansen, Lars Hestbjerg Hansen, John Mortensen, Per Meyer Jepsen

**Affiliations:** 1 Roskilde University, Department of Science and Environment, Roskilde DK-4000, Denmark; 2 Aarhus University, Department of Environmental Science, Roskilde DK-4000, Denmark

**Keywords:** Copepod, DHA, Biosynthesis, Gene expression, Transcriptome

## Abstract

The cyclopoid copepod *Apocyclops royi* (Lindberg 1940) is one of two dominant mesozooplankton species in brackish Taiwanese aquaculture ponds. Periodically low n-3 polyunsaturated fatty acid (PUFA) content in seston could potentially be a limiting factor for zooplankton diversity. *Apocyclops royi*’s potential ability to biosynthesize n-3 PUFA was investigated through a short-term feeding experiment on four species of microalgae*.* Furthermore, we analyzed the expression of genes encoding putative fatty acid elongase (*ELO*) and desaturase (*FAD*) enzymes in *A. royi* on long-term diets of the PUFA-poor *Dunaliella tertiolecta* and the PUFA-rich *Isochrysis galbana*. The copepods exhibited high contents of docosahexaenoic acid (DHA, C22:6n-3) (>20% of total fatty acid) even when DHA-starved for two generations, and no significant differences were found in absolute DHA content between treatments. Transcripts correlating to the four enzymes Elovl4, Elovl5, Fad Δ5 and Fad Δ6 in the n-3 PUFA biosynthetic pathway were identified. Gene expression analysis revealed a significantly higher expression of two desaturases similar to Fad Δ6 in copepods fed PUFA-lacking algae compared to copepods fed algae with high PUFA content. These findings suggest a highly active n-3 PUFA biosynthesis and capability of DHA production in *A. royi* when fed low-PUFA diets.

## Abstract (precis)

**Summary:** Here, we demonstrate n-3 PUFA biosynthesis in the copepod *Apocyclops royi* from Taiwanese aquaculture pond systems using fatty acid profiling and gene expression analysis.

## INTRODUCTION

The tropical climate of Taiwan gives rise to abundant zooplankton communities in coastal waters (Dur et al., 2007; Hwang et al., 2003; Ju et al., 2018), rivers and estuaries (Hwang et al., 2010; Beyrend-Dur et al., 2013). Even highly eutrophicated lagoons show rich zooplankton biodiversity (Lo et al., 2004). On the other hand, a case study has shown that adjacent man-made brackish aquaculture ponds in which copepods are reared as live feed for fish larval production reveal a rather simplistic zooplankton community (Blanda et al., 2015). This is despite periodical inoculation of zooplankton from nearby species-rich estuaries (Rayner et al., 2015; Yu, 2004). The ponds are approximately 1 ha in size, with a depth of 1 m and very often with an oily and foamy water surface (see Blanda et al., 2015 for details). The main limiting factor for overall copepod abundance in the ponds was found to be the poor water quality, including frequent severe hypoxia events as low as 0.7 mg l^−1^ during the night (Blanda et al., 2015).

Two species of copepods dominate the aquaculture ponds: the calanoid *Pseudodiaptomus annandalei* and the cyclopoid *Apocyclops royi* (Blanda et al., 2015, 2017; Su et al., 2005). This community reduction raises the question of whether the two species have crucial traits in common in order to occupy the ecological niche of the environmentally harsh conditions of this artificial habitat. The common trait could be tolerance to extreme high temperature or hypoxia, or the driving force could be the fluctuating availability of nutritious food.

In a summer study in 2012 and a year-long study in 2013–14, it was reported that the copepods were experiencing prey *ad libitum* (Blanda et al., 2015, 2017). Phytoplankton densities were high, and the seston concentration was a minimum of 7540±1630 µg C l^−1^ in 2012 and an estimated minimum of 3000 µg C l^−1^ in 2013–2014. The carbon to nitrogen (C:N) ratio of the seston was 6.4±0.3 in 2012, and the overall quality of the seston was considered adequate for zooplankton production (Blanda et al., 2015). In a study by Rayner et al. (2015), discrepancies were found between relatively poor seston and relatively richer *P. annandalei* fatty acid (FA) profiles, especially regarding high copepod levels of C18:1n-9 and DHA, both endpoints of FA syntheses. C18:1n-9 is often considered a trophic marker for omni- and carnivorous copepods (Dalsgaard et al., 2003). The authors proposed that *P. annandalei* was either selectively feeding on the more nutritious fish/shrimp meal added to the pond system or the copepods was further metabolising C16:0 and α-linolenic acid (ALA, C18:3n-3) (Rayner et al., 2015). Blanda et al. (2017) reported that in 2013–2014 the same discrepancies in content of C18:1n-9 was not observed, while it was still present for DHA. The lack of C18:1n-9 suggested a phytoplankton-based diet, but this could not account for the observed high DHA content in the copepods. The relatively large amount of DHA in *P. annandalei* compared to seston could suggest bioconversion of ALA to DHA (Rayner et al., 2017). DHA and the precursor eicosapentaenoic acid (EPA, C20:5n-3) are both important for the fecundity of copepods (Jónasdottir, 1994; Evjemo et al., 2008; Støttrup and Jensen, 1990). The ability to produce EPA and DHA in environments with PUFA-low seston could enable *P. annandalei*’s and *A. royi*’s survival in the ponds.

Blanda et al. (2017) reported that the PUFA content in seston was low all year round (23.2–34.4% of total FA content). The essential FAs – EPA and DHA – were especially low during spring, summer and fall (<10% each). However, *P. annandalei* consistently had a FA profile with higher PUFA content (33.5–74.1%), and even reached a DHA content of 46.6% of total FA in January 2014. Furthermore, Blanda et al. (2017) reported that the mean DHA content in the seston was as low as 2.0±1.1% of total FA during a July/August campaign. Considering the high metabolic rate in tropical organisms, this suggests that occasionally the copepods are PUFA-starved.

Rayner et al. (2017) argued that *P. annandalei* is able to further metabolize ALA into n-3 long-chain polyunsaturated fatty acids (LC-PUFA). The pathway of the bioconversion of n-3 LC-PUFA is well described in the literature (Monroig et al., 2013; Sprecher, 2000; Oboh et al., 2017), however proof of the actual ability is seldom provided in studies of marine invertebrates. Other copepod species are also likely to possess the ability for n-3 LC-PUFA biosynthesis. This ability is suggested to be present in the calanoids *Calanus finmarchicus* (Bell et al., 2007) and *Paracalanus parvus* (Moreno et al., 1979), the harpacticoid *Tisbe holothuriae* (Norsker and Støttrup, 1994), the cyclopoids *Eucyclops serrulatus* (Desvilettes et al., 1997) and *Paracyclopina nana* (Lee et al., 2006), and also in the candidate species of the present study *A. royi* (Pan et al., 2017). These studies used direct comparison of microalgae diet and copepod FA profiles to support their claims, except Bell et al. (2007) and Moreno et al. (1979) who used another approach and conducted isotope-marking experiments that made it possible to directly follow the progression of the fatty acid bioconversion and gives more substantial proof of biosynthesis. However, even if the copepods possess the ability of fatty acid bioconversion, hitherto the quantitative analyses have illustrated very low levels of biosynthesis (Bell et al., 2007; Moreno et al., 1979).

Another approach to provide indications of fatty acid bioconversion is by analysing the gene expression of copepods. In vertebrate FA biosynthesis, seven enzymes have been identified to be responsible for the bioconversion of PUFA; the elongases Elovl 2, 4 and 5, and the desaturases Δ 4, 5, 6 and 8. The genes for these enzymes are all well known, and similar genetic patterns have been found in several marine invertebrate species (Monroig et al., 2013; Surm et al., 2015). In marine invertebrates, the analysis of genes encoding Δ5 and Δ6 *FAD*s has proven difficult, as a bifunctionality is often observed, and as Δ5 and Δ6 *FAD* sequences do not seem to form distinct clades, but rather are intermixed (Monroig and Kabeya, 2018; Kabeya et al., 2018; Wu et al., 2018). For copepods however, the knowledge on FA synthesis pathways is very limited, making comparison of both gene expression and gene similarity difficult. It has previously been noted that larger databases would allow for more thorough studies of copepod physiological responses to their environments (Bron et al., 2011).

We hypothesize that a long-term deficit in access to n-3 LC-PUFA can promote the n-3 PUFA biosynthetic pathway in *A. royi*. This may be the main limiting factor leading to success for only two copepod species in the Taiwanese fish ponds. The purpose of the present study is therefore to illustrate to what extent FA modifications take place in a key species of copepods, *A. royi*, in an environment with PUFA-poor seston. Therefore, we pursue the idea of demonstrating (i) the ability of *A. royi* to synthesize DHA in large quantities, and (ii) a selective activation of the gene apparatus in *A. royi* promoting n-3 LC-PUFA bioconversion. Further, we provide information on potential n-3 PUFA related desaturase genes from ten copepod species, including the candidate species of the present study, *A. royi*, as well as the cyclopoids *E. serrulatus* and *P. nana*, the calanoids *C. finmarchicus* and *Neocalanus flemingeri*, the harpacticoids *T. holothuriae*, *Tigriopus japonicus* and *Tigriopus californicus*, and the Siphonostomatoida *Caligus rogercresseyi* and *Lepeophtheirus salmonis*.

## RESULTS

### Fatty acid analyses

Algae samples were labelled with species name while copepod samples were labelled with abbreviations of the algae diet, i.e. DUN, ISO, RHO and TET. ISO did not reproduce sufficiently to continue to the second generation. Therefore, copepod FA analysis was not done for the ISO treatment.

The FA profiles of the algae differed from each other, especially regarding n-3 PUFA (Table 1). *Dunaliella tertiolecta* had the highest content of ALA (60.20±2.51%) and the lowest content of stearidonic acid (SDA, C18:4n-3; 1.29±0.22%) compared to the other algal species. Furthermore, it lacked EPA and DHA. *Isochrysis galbana* had the highest content of DHA (28.37±1.70%), but low content of ALA, SDA and EPA (3.70±0.15, 4.95±0.30 and 0.90±0.08%). *Rhodomonas salina* had the highest content of SDA (25.80±0.93%) and overall high contents of ALA, EPA and DHA (21.14±0.76, 15.22±0.92 and 9.42±0.23%). *Tetraselmis suecica* had the highest content of EPA (19.47±1.63%) but lacked DHA.

**Table 1.**
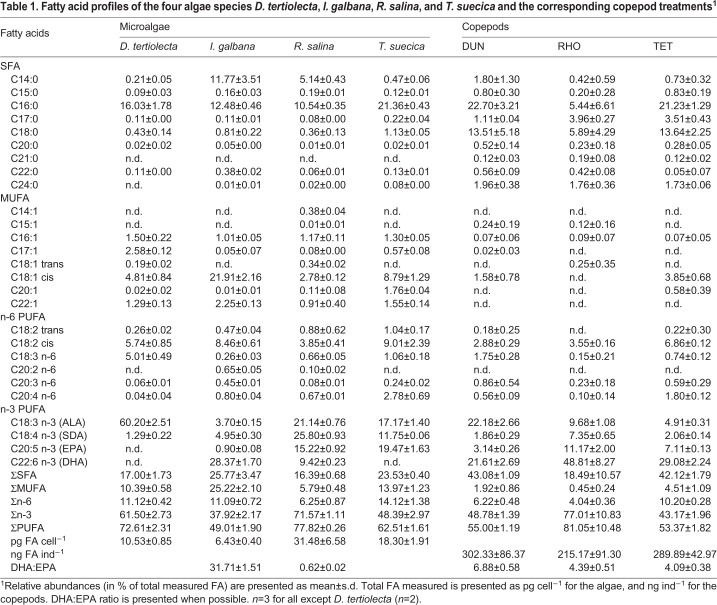
**Fatty acid profiles of the four algae species *D. tertiolecta*, *I. galbana*, *R. salina*, and *T. suecica* and the corresponding copepod treatments^1^**

**Fig. 1.**
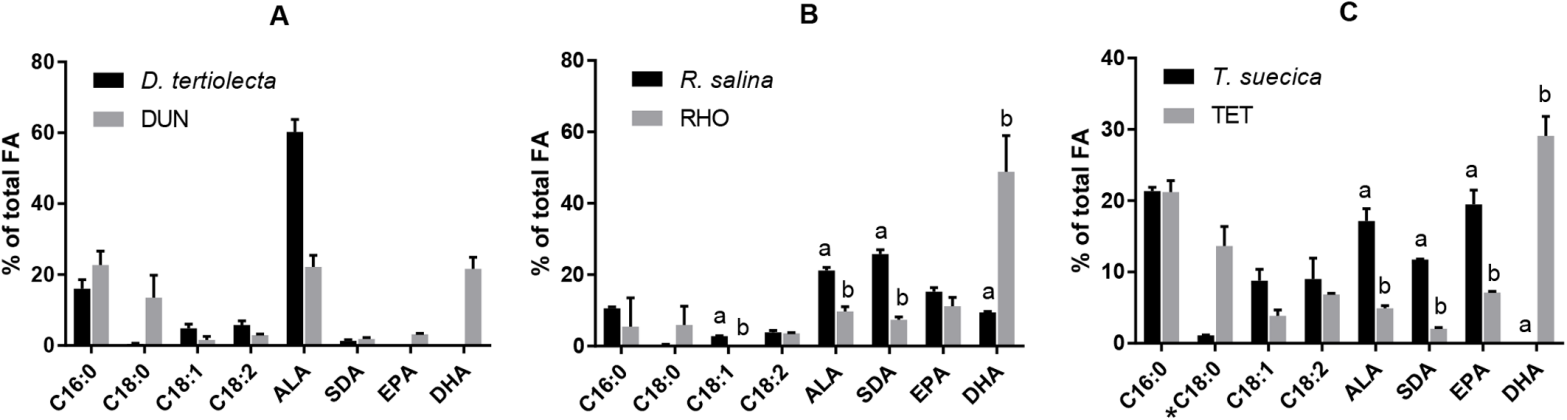
**Comparison of the relative content of n-3 LC-PUFA and their immediate precursors in algae and copepods.** Samples of 20 individuals of *A. royi* or approximately 500 µg C of the algae species were collected from triplicate treatments. The columns represent mean±s.d., *n*=3 for all except *D. tertiolecta* (*n*=2), statistics were not performed for A. *t*-tests were performed for all comparisons expect for non-parametric datasets (*), for which Mann–Whitney *U*-tests were performed. Significant differences are denoted with letters (*P*<0.05).

The FA profiles of the copepods differed depending on diet. The FA profiles of algae and copepods were compared and are presented in Fig. 1. For comparison of *D. tertiolecta* and DUN statistics were not performed as *n*=2 for *D. tertiolecta*. DUN exhibited lower ALA content than *D. tertiolecta,* 60.20±2.51 and 22.18±2.66%, respectively. DUN also contained 3.14±0.26% EPA and 21.61±2.69% DHA despite these two FAs were not detected in its diet. RHO had significantly lower contents of ALA and SDA (9.68±1.08 and 7.35±0.65%) compared to *R. salina* (21.14±0.76 and 25.80±0.93%), *P*<0.01. Furthermore, the DHA content was significantly higher in the RHO treatment (48.81±8.27%) than in the algae (9.42±0.23%), *P*<0.01. TET had significantly lower contents of ALA, SDA and EPA (4.91±0.31, 2.06±0.14 and 7.11±0.13%) compared to *T. suecica* (17.17±1.40, 11.75±0.06 and 19.47±1.63%), *P*<0.001. Similar to the DUN treatment, TET exhibited high contents of DHA (29.08±2.24%) even though it was absent in the copepods diet. For RHO and TET treatments, no significant differences (*P*>0.05) were found between algae and copepods in the biosynthetic precursors of ALA; C16:0, C18:0 and C18:2n-6. The C18:1n-9 content was not significantly different between TET and *T. suecica*, and it was not found in the RHO treatments whilst it was present in *R. salina.*

**Fig. 2.**
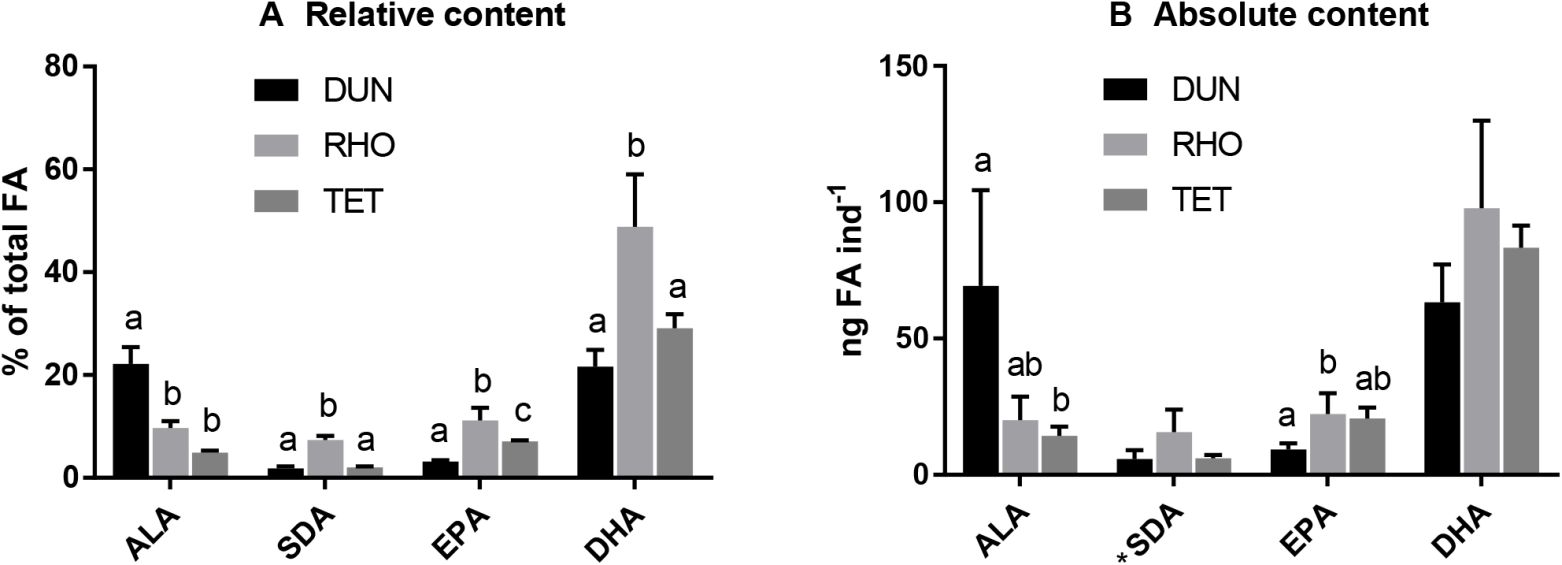
**Comparison of n-3 PUFA content between copepod treatments.** Samples of 20 individuals of *A. royi* were collected from triplicate treatments. Columns represent mean±s.d., *n*=3. (A) Relative content presented by % of total FA. (B) Absolute content presented by ng ind^−1^. One-way ANOVA and Tukey's test were performed for all datasets except for non-parametric datasets (*), for which Kruskal–Wallis and Dunn's tests were performed. Significant differences are denoted with letters (*P*<0.05).

The present study did not determine diet-induced differences in fecundity and can therefore not use reproduction as an estimate of whether DHA production was adequate. Therefore, not only the relative contents but also the absolute contents of n-3 PUFA in the copepod treatments were compared (Fig. 2). DUN had significantly higher relative contents of ALA than the TET and RHO treatments (*P*<0.01). RHO had significantly higher relative contents of SDA, EPA and DHA (*P*<0.05) compared to the others. DUN and TET did not have significantly different relative contents of DHA, but TET had significantly higher relative EPA content than DUN (*P*<0.05).

DUN had significantly higher absolute content of ALA compared to TET (69.35±28.63 and 14.33±2.72 ng ind^−1^), *P*<0.05, but RHO was not significantly different from either of them (20.0±7.08 ng ind^−1^). RHO had significantly higher absolute content of EPA compared to DUN (22.33±6.2 and 9.27±1.85 ng ind^−1^), *P*<0.05, but TET was not significantly different from either of them. However, no significant differences were found between treatments in the absolute values of SDA and DHA. Absolute DHA contents of DUN, RHO and TET were 63.36±11.24, 97.83±26.22 and 83.33±6.61 ng ind^−1^, respectively.

### Genetic analysis

Reads from all eight replicate mRNA samples were mapped to the transcriptome assembly GHAJ01. The samples consisted of an average of 24.7±7.9 M reads. In all samples, just over half of the reads aligned to the transcriptome assembly (average 53±1.1%). The cause of the relatively low aligning percentage of reads is the length cutoff for transcripts of 500 bp, as >85% of reads align to the full dataset (data not shown). We believe that this cut-off does not affect the analysis of *FAD*s and *ELO*s as all PFAM SEED sequences in the two families are longer than 500 nt.

As the cDNA data is stranded and complimentary to the mRNA, only reads mapping in the reverse direction were counted. Between 96.1% and 97.2% of mapped reads were mapped in the reverse direction in all samples, demonstrating a successful stranded mRNA sequencing library preparation (data not shown). Read normalization was performed in CLC genomics 11.0, utilizing the TMM (trimmed mean of M values) normalization method (Robinson and Oshlack, 2010).

In total 12 transcripts from the *A. royi* mRNA dataset GHAJ01 was found to be likely *FAD* Pfam family members (PF00487) and 10 belonged to the *ELO* Pfam family (PF01151). Of the identified genes, 13 were annotated to potentially participate in the n-3 PUFA biosynthetic pathway (Fig. 2). Of the n-3 PUFA related *FAD*s, six transcripts were reassembled into three genes (alignment between GHAJ01 sequences and complete genes can be found in Fig. S1 and the complete nucleotide sequence of the complete genes can be found in Table S1). We found transcripts coding for predicted functions similar to the four enzymes Elovl4, Elovl5, Fad Δ5 and Fad Δ6 (Table 2). Elovl5 is associated with elongation of C18 to C20 and C22, and Elovl4 is usually associated with elongation of C24 up to C36, while it has shown elongation of C20 to C22 and C24 in some species of fish (Monroig et al., 2013). Fatty acid desaturase nomenclature indicates the site of the resulting double bond, i.e. Fad Δ5 removes protons from the C_5_–C_6_ position and Fad Δ6 removes protons from the C_6_–C_7_ position. Fad Δ6 is associated with desaturation of ALA to SDA and C24:5n-3 to C24:6n-3., while Fad Δ5 is associated with desaturation of C20:4n-3 to EPA. It is difficult to predict if an invertebrate Fad performs Δ5 or Δ6 desaturation, as an overlap in function has been reported and because the genes performing the functions could be the result of convergent evolution rather that shared ancestry (Sperling et al., 2003; Kabeya et al., 2018). Other genes associated with elongation and desaturation were found but were left out of this study as they were not deemed relevant for n-3 LC-PUFA synthesis, or not specifically annotated with a relevant function.

**Table 2.**
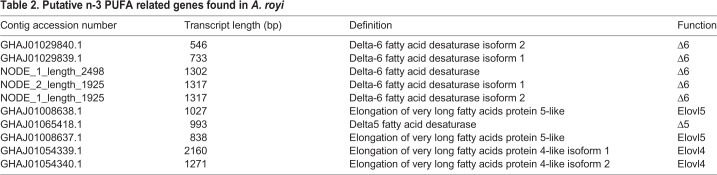
**Putative n-3 PUFA related genes found in *A. royi***

#### Differential expression analysis of n-3 PUFA related FADs and *ELO*s

For 2 months (∼7–8 generations), two cultures of copepods were fed *I. galbana* and *D. tertiolecta*, respectively. The copepods of both cultures were lively and reproducing. Samples of copepods fed *I. galbana* were named I1–I4, and samples of copepods fed *D. tertiolecta* were named D1–D4. We chose to exclude the sample I2 from analysis because it had lower frequency of aligning reads than the other samples and because the sample dominated the PCA plot when included (data not shown). This way, a total of three samples from animals fed *I. galbana* and a total of four samples fed *D. tertiolecta* were used for differential expression analysis.

**Fig. 3.**
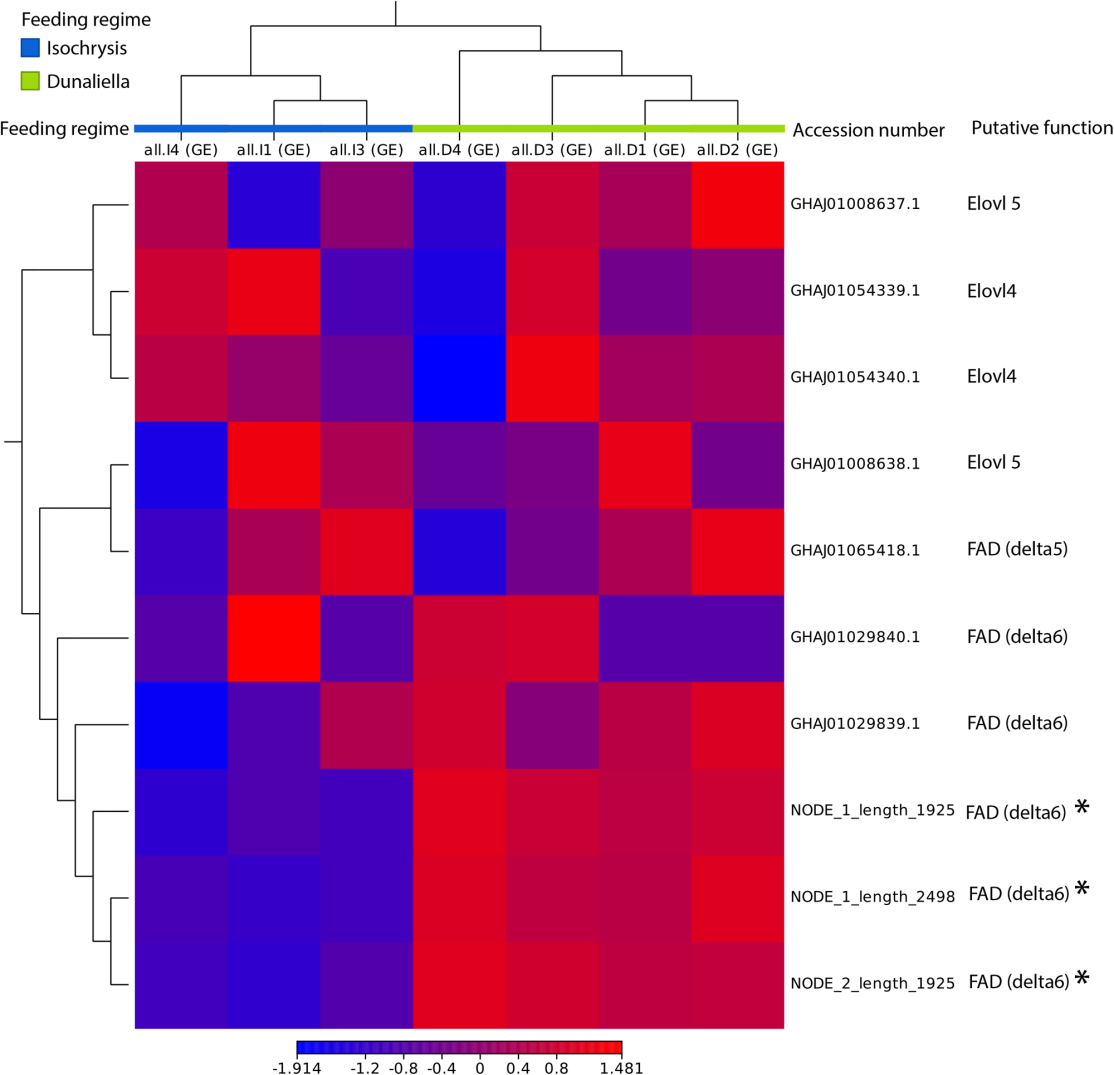
**Heatmap of putative n-3 PUFA related Elovls and Fads found in *A. royi* fed *D. tertiolecta* and *I. galbana*.** *t*-test were performed for each gene. * denotes significant difference (*P*<0.000001) in gene expression of the two differently fed *A. royi* treatments. Note that only the genes NODE_1/2_length_1925 and NODE_1_length_2498 are differentially expressed and that the genes are upregulated in animals fed the PUFA-poor algae *D. tertiolecta* compared to animals fed the PUFA-rich *I*. *galbana*.

Comparing the gene expression between the two cultures of copepods, three putative n-3 PUFA related desaturases are significantly more expressed in copepods fed *D. tertiolecta* than copepods fed *I. galbana* (*P*<0.000001). Two of the three differentially expressed *FAD*s are isoforms of each other and share >99% of the amino acid sequence. No significant difference in expression between feeding regimes was seen for genes putatively encoding Elovl4, Elovl5 or three other putative n-3 PUFA related desaturase genes (Fig. 3). These findings indicate an n-3 PUFA starvation induced expression of two potentially rate-limiting LC-PUFA related desaturases.

#### Phylogenetic placement of differentially expressed n-3 PUFA related desaturase genes

**Fig. 4.**
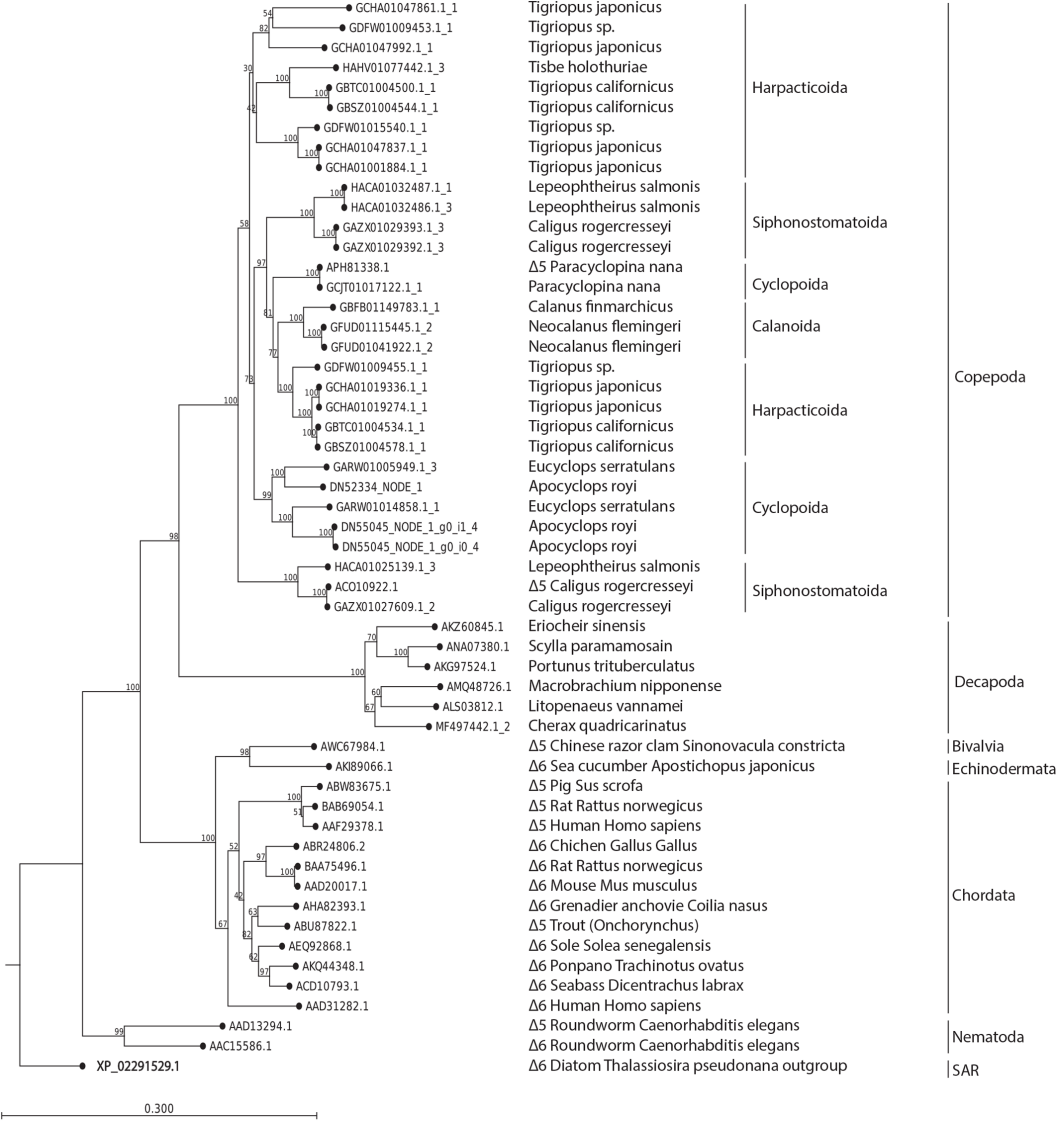
**Neighbor joining tree of 29 identified copepod FA desaturase genes and an additional 19 reported animal Δ5 and Δ6 FA desaturase genes.** The copepod sequences form a single clade, which is a sister clade to the six decapod sequences. These are again a sister clade to the remaining animal sequences from Nematoda, Bivalvia, Echinodermata and Chordata. The diatom *Thalassiosira pseodonana* was used as an outgroup. Note that within the copepod clade, the identified genes cluster according to order, with two clades from each of the orders Harpacticoida, Cyclopoida and Siphonostomotioda, while all Calanoida sequences are found in one clade. Potentially, this spilt could be related to the function of gene products. There is good support for all deep branches, while several branchings – e.g. between the copepoda orders – have low bootstrap values and are thus not reliable, while the bootstrap values are very high within the copepod orders.

In order to understand the function of the food-dependent differentially expressed desaturase genes, we aligned them to other animal desaturases. This could potentially explain the function of the gene products, and by identifying similar genes in a wide range of copepod datasets, we further justify the accuracy of the gene sequence and demonstrate the conserved nature of these genes within Copepoda. In Fig. 4, a neighbor-joining tree of 29 identified copepod n-3 PUFA related desaturase genes, six decapod desaturase genes and 19 desaturase genes from a range of animals can be seen. These comprise, to the best of our knowledge, the most complete overview of copepod desaturases to date. Interestingly, all copepod sequences are found in a single clade, and all decapod sequences similarly make up a distinct clade. Further, both Fad Δ5 and Δ6 genes from chordates make up a single clade, with a sister clade consisting of a bivalve Fad Δ5 and an echinoderm Fad Δ6. A sister clade to all sequences seem to be two *Caenorhabditis elegans* genes encoding Fad Δ5 and Δ6. Notably, the two annotated copepod desaturases APH81338.1 (Δ5, *P. nana*) and ACO10922.1 (Δ5, *C. rogercresseyi*) were also identified by our BLAST+HMM+B2G TSA pipeline. In Fig. 4, the identified copepod genes form distinct clades that fit the orders Calanoida, Harpacticoida, Cyclopoida and Siphonostomatoida, although the latter three each occupy two clades in the tree. The complete amino acid sequence and accession numbers of the identified copepod genes can be found in Table S2.

## DISCUSSION

### Fatty acid analysis

*Apocyclops royi* was fed four different microalgae and to compare between copepods and their diet FA analysis were performed on the microalgae species. For *D. tertiolecta* a rather high amount of ALA was detected (>60% of total FA measured). It cannot be excluded that some quantitatively important FAs were not included in the analysis. For *D. tertiolecta* both Volkman et al. (1989) and Delaunay et al. (1993) reported high values of the FA C16:4n-3, which has not been measured in the present study. Both studies found a ratio of ALA:C16:4n-3 of approximately two. Assuming the same ratio was present in the *D. tertiolecta* of the present study, it is likely that the actual ALA content was approximately 46.3% of total FA, which is rather close to the 43.5% of total FA reported by Volkman et al. (1989). Therefore, it was assumed that the results of the present study are representative.

*Apocyclops royi* was fed four different microalgae, two of which lacked DHA and one of those algae furthermore lacked EPA. Despite this, EPA and DHA was found in all copepod treatments (on which FA analysis were performed), suggesting an active n-3 LC-PUFA synthesis in *A. royi*. This was further supported by the smaller relative contents of ALA, SDA and EPA found in RHO and TET, indicating that the biosynthetic process was progressing. These overall findings support the findings of Pan et al. (2017).

Despite being DHA starved for two generations *A. royi* fed *D. tertiolecta* or *T. suecica* were still exhibiting high contents of DHA (>20%). This indicates that the n-3 PUFA biosynthesis was highly active, and the fact that the absolute DHA contents were not significantly different between diet treatments suggests that *A. royi*, unlike most species, is able to produce adequate amounts of DHA to cover its own physiological functions sufficiently to maintain its population. This is not further investigated in the present study, e.g. in the form of a fecundity analysis, which would be beneficial as n-3 LC-PUFA is important for egg production (Jónasdóttir, 1994; Evjemo et al., 2008; Støttrup and Jensen, 1990). However, Pan et al. (2017) reported no significant differences in fecundity between *A. royi* fed *I. galbana* and copepods fed the DHA-lacking *Tetraselmis chuii* for 14 days (∼two generations). This suggests that while DHA biosynthesis is energy consuming, the trade-off in form of access to n-3 LC-PUFA for reproduction is profitable. The effect of PUFA starvation and subsequent DHA biosynthesis could be further investigated by comparing the expression of stress and reproduction related genes of copepods fed diets with high and low amounts of DHA.

Despite Pan et al. (2017) reporting high fecundity for *A. royi* fed *I. galbana*, the ISO treatment copepods of the present study did not reproduce sufficiently to continue the second generation. Lee et al. (2006) found that for the cyclopoid copepod *P. nana* somatic growth was slower for copepods fed *I. galbana* in contrast to copepods fed *T. suecica*. Furthermore, *A. royi* fed solely on *I. galbana* was kept successfully for 2 months for the present gene expression experiment. Therefore, it was assumed that maturation time was simply underestimated for the present FA analysis experiment. The cause was however not further investigated here.

### FAD-like and ELO-like transcript identification

Transcripts coding for putative desaturases and elongases enzymes were found using a trio BLAST-HMM–B2G approach using the Pfam seed databases of *desaturase* and *elongase* genes. Several transcripts were found in *A. royi* that matched genes for elongases and desaturases, but only transcripts functionally annotated by B2G as relevant to the n-3 PUFA biosynthesis were further investigated. Transcripts similar to four enzymes out of seven relevant were found: Elovl4, Elovl5, Fad Δ5 and Fad Δ6. Transcripts coding for the three related enzymes Elovl2, Fad Δ4 and Fad Δ8 were not found. Fad Δ4 and Fad Δ8 are relatively rare and have presently mostly been found in vertebrate species (Morais et al., 2012; Li et al., 2010; Monroig et al., 2011). Furthermore, commonly only two Elovl families are found in invertebrates: Elovl4 and a single Elovl5/2-like protein that covers the functionality of both Elovl2 and Elovl5, the latter of which has been found present in the copepod *C. rogercresseyi* (Monroig et al., 2013). It is considered likely that an Elovl4 or Elovl5/2 will cover the Elovl2 functions in crustaceans (Monroig and Kabeya, 2018). Assuming the Elovl2 functions are covered, the four enzymes together with β-oxidation can account for the entire biosynthesis of ALA to DHA according to the Sprecher pathway (Oboh et al., 2017; Sprecher, 2000; Monroig et al., 2013), Fig. 5. Therefore, we find it possible that *A. royi* utilizes a four-enzyme system for converting ALA to DHA. The same enzymes are responsible for the n-6 PUFA synthesis. However, the n-6 products are found in only neglectable amounts in *A. royi* (<2% of total FA each) and are thus not further discussed in this study.

**Fig. 5.**
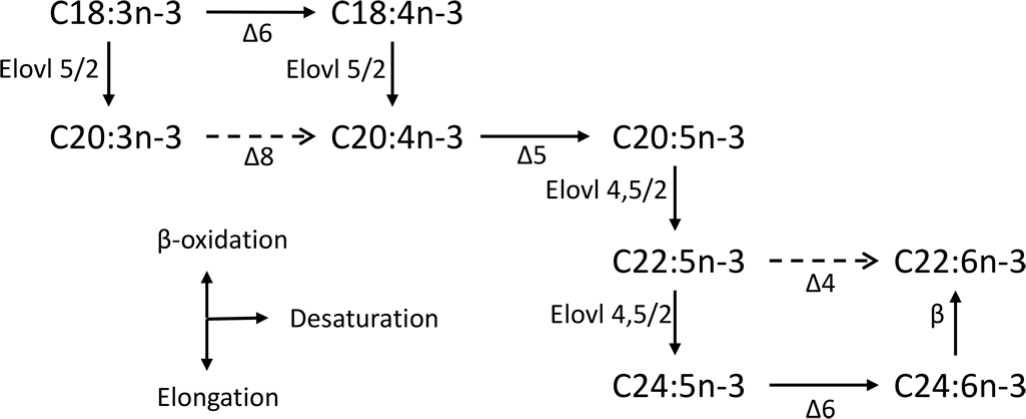
**Biosynthetic pathway proposed by** **Sprecher (2000)** **within the capabilities of *A. royi*.** Solid arrows indicate pathways where corresponding genes were found. Dashed arrows indicate alternative pathways not presently found in *A. royi*. Horizontal arrows indicate desaturation, vertical arrows pointing downwards indicate elongation, vertical arrows pointing upwards indicate β-oxidation.

### Differential n-3 PUFA related gene expression based on feeding regime

The purpose of this experiment was to imitate high versus low quantity PUFA seston to investigate whether the possible high tolerance of *A. royi* to low PUFA availability was due to an inducible n-3 PUFA biosynthesis. For the differential expression analysis, we have used the CLC Genomics RNA-seq module, which in a recent study was found to be among the most accurate performers in a test of 14 common RNA-seq analysis pipelines. The benchmarking also highlighted the robustness of the CLC Genomics pipeline when using default parameters (Baruzzo et al., 2017). *I. galbana* was chosen as feed in the gene expression experiment because of its high DHA content (28.37±1.70%), potentially diminishing the need for n-3 PUFA biosynthesis. *D. tertiolecta* was chosen as feed because of its lack of EPA and DHA, potentially activating the n-3 PUFA biosynthesis. In order to obtain a pure animal sample, it was necessary to cold sediment the animals at 0°C to remove particular matter such as dead animal parts and fecal pellets and suspended particles such as ciliates and bacteria. Furthermore, the cooled seawater was of a higher salinity (32 psu) than the culture water the animals were cultured in (20 psu). This treatment lasted for approximately 1 h and could potentially have an effect on the subsequent results. However, both animals fed *I. galbana* and animals fed *D. tertiolecta* were handled similarly, so the observed differences in gene expression is unlikely to stem from the cold sedimentation or salinity changes, though there might be differences between our mRNA data and the expression profile in animals which were not cold sedimented.

Significant differences were found only in the expression of three desaturase like transcripts, which possess the typical front-end desaturase like domain cytochrome b5 (containing the sequence HPGG) followed by three Histidine motifs [HDANH, HVVQHH and QIEHH, respectively (Monroig and Kabeya, 2018; Sperling et al., 2003) (Figs 3 and 5), nucleotide sequences can be found in Fig. S1. For these three complete genes, *D. tertiolecta* fed copepods had a significantly higher expression level (*P*<0.0000001)]. While the phylogenetic analysis seen in Fig. 4 did not determine if the function of the desaturases with PUFA dependent expression is Δ6 or Δ5, the genes are very similar to a large family of mixed Δ5 and Δ6 desaturases. Fad Δ6 is specifically responsible for desaturation of ALA to SDA and C24:5n-3 to C24:6n-3, the first and final steps of desaturation in the n-3 PUFA biosynthetic pathway, and thus a key enzyme in n-3 PUFA biosynthesis. Desaturation is a more energy-costly step than elongation (Bell et al., 2007), and especially Fad Δ6 is considered rate limiting in the biosynthetic pathway (Gregory et al., 2011). Therefore, the increased frequency of transcripts mapping to genes for a putative Fad Δ6 desaturase in *D. tertiolecta* fed copepods compared to *I. galbana* fed copepods demonstrates that n-3 PUFA-poor seston will induce the n-3 PUFA biosynthetic pathway and thereby catalyze the production of these essential FAs. In order to confirm these results, it would be necessary to clone and express the genes to classify them by substrate.

Transcripts similar to the other three enzymes (Fad Δ5, Elovl4 and Elovl5) were not found to have a significantly different expression profile between feeding regimes. Overall, genes potentially coding for the entire Sprecher biosynthetic pathway was found in both the *I. galbana* and *D. tertiolecta* fed copepods. The biosynthesis is more active in copepods fed low-PUFA diets but is still active in copepods fed high-PUFA diets.

The conception that copepods are incapable of n-3 PUFA biosynthesis (Dalsgaard et al., 2003) has been challenged as several species of copepods have been argued to possess the capability, even across orders (Bell et al., 2007; Desvillettes et al., 1997; Lee et al., 2006; Pan et al., 2017; Norsker and Støttrup, 1994; Moreno et al., 1979). This raises the question of whether the ability is a common trait of pelagic copepods that is simply downregulated in some species. This question ought to be pursued by a long-term study including several copepods species.

However, there are differences in the biosynthetic pathways proposed for *A. royi* in the present study and *P. annandalei* (Rayner et al., 2017). Rayner et al. (2017) proposed that *P. annandalei* elongates ALA to C20:3n-3 and thereafter desaturates it to C20:4n-3, i.e. the Δ8-pathway, while the present study has not found evidence of this pathway for *A. royi*. However, when only assessing the FA content and not the gene expression and enzyme functions, the results can be misleading. This is because Elovl5 is involved in elongation of both ALA and SDA, and therefore the elongation of ALA could lead to a pooling of C20:3n-3 if the copepod lacks Fad Δ8 (Monroig et al., 2011). Simply producing a FA does not mean that the n-3 PUFA synthetic pathway is through that specific FA. Only assessing FA content could lead to false conclusions of the presence of Fads such as Δ8 but also Δ4 when investigating the path from C22:5n-3 to DHA. Therefore, we suggest that gene expression analysis should also be performed for *P. annandalei* to properly investigate whether the periodically low PUFA content of the copepod food particles in the fish ponds is the limiting factor behind the simplistic zooplankton community. Furthermore, while *A. royi* had relatively high contents of DHA (>20%), *P. annandalei* only had (5.4%). This could suggest that *A. royi* is even more flexible in its DHA biosynthesis (and may even produce DHA in excess) compared to *P. annandalei*. A comparative study involving isotope-marked ALA tracing experiments and fecundity could quantify not only PUFA biosynthesis efficiency but also sufficiency.

### The origin of differentially expressed FAD-like transcripts

Initial efforts to extract FA desaturases from databases and build phylogenies to place the putative *A. royi* FA desaturases failed because of the scarcity of annotated sequences and because of the variability of *FAD* genes, why a more thorough copepod FA desaturase identification based on protein sequences was performed.

In order to phylogenetically place the differentially expressed desaturase-like sequences and to understand the relationship between them, we established a database of genes from other copepod species for comparison. Using the same trio BLAST+HMM+B2G approach used for initial identification of *A. royi FAD*s and *ELO*s, we searched the existing 22 copepod TSAs from 16 copepod species. The resulting sequences were annotated using B2G with the same workflow and parameters as for *A. royi*, and the genes were then manually curated to obtain a selection of 26 highly similar genes to the differentially expressed *A. royi* desaturases from 10 species covering the four most ecologically important copepod orders of Cyclopoida, Calanoida, Harpacticoida and Siphonostomatioda. All reading frames of the identified copepod desaturase-like genes were translated and the correct reading frame was manually identified by searching for the conserved invertebrate desaturase motifs HPGG (cytochrome b5), and the three identified histidine boxes HDXXH, HVVQHH and H/QXXHH. The amino acid sequence of the three differentially expressed *A. royi* genes, 26 identified copepod desaturases and 17 other likely front-end desaturases from a wide range of animals was aligned and a neighbor-joining phylogenetic tree of the sequences was constructed using CLC11.0 (Distance measure=Jukes-Cantor, Bootstrap=1.000 Replicates). A list of the sequences and the correct reading frame can be found in Table S3, the alignment can be found in Fig. S2. Within the chordate clade in the tree, it is remarkable how the Fad Δ5 and Δ6 genes do not form distinct clades, but rather are intermixed. Similarly, the *C. elegans* Fad Δ5s and Δ6s are closest to each other, rather than to Fad 5Δs and Δ6s from other species. This could indicate that the speciation of organisms in Fig. 4 is older than the split between the function of Fad Δ5 and Δ6 genes, which has also previously been reported (Sperling et al., 2003). That the decapod sequences which form a sister clade to copepods are annotated as Fad Δ6s does similarly not mean that they perform this function only: the identified enzymes have not been experimentally demonstrated to be Fad Δ6. Because of the difficulties of bioinformatically demonstrating the function of FA desaturases, and because of the scarcity of information on copepod genetics, we are not confident that the differentially expressed genes seen in the heatmap in Fig. 3 are Fad Δ6s or Fad Δ5s. Rather, we are confident that the identified copepod sequences belong to a large family of conserved FA desaturases similar to the previously reported front-end desaturases, which are necessary and sufficient for the desaturation in invertebrate n-3 PUFA biosynthesis.

### Closing remarks

The PUFA-poor environment in Taiwanese aquaculture ponds are for periods low in available n-3 PUFA content in seston, possibly limiting the diversity of the zooplankton community. The present study has illustrated that one of the two dominant copepod species, *A. royi*, is capable of adjusting its metabolic activity of n-3 PUFA biosynthesis in periods of PUFA starvation. This flexibility in DHA production has to our knowledge not been illustrated in other zooplankton species, and this flexibility likely gives *A. royi* a change of survival in highly variable aquaculture ponds. This should be further investigated through feeding and fecundity experiments on other local species, but as the other dominant species, *P. annandalei* also shows strong indications of this capability (Rayner et al., 2017), it supports the importance of inducible n-3 PUFA biosynthesis capabilities in respect to survival in the fish ponds.

## MATERIALS AND METHODS

### Stock cultures

#### Algae cultures

The four marine microalgae species selected for this experiment, *D. tertiolecta* (K-0591), *I. galbana* (K-1355), *R. salina* (K-1487) and *T. suecica* (K-0949), were kept as pure strains at Roskilde University, Denmark*.* These species were chosen because of their different FA profiles, especially concerning n-3 PUFA.

The batch cultures were kept in triplicate 1 l round-bottom flasks under identical conditions. They were cultivated in 30 psu 0.2 µm UV filtrated seawater at 17°C, with aeration, and continuous 50–65 µmol PAR photons m^−2^ s^−1^. Nutrition was administered daily in the form of modified f/2 medium (Guillard, 1975, without cobalt *sensu* Thoisen et al., 2018). Cell density was maintained daily at the exponential growth phase to ensure nutritional homogeneity within each algae species.

#### Copepod cultures

*A. royi* was obtained from Tungkang Biotechnology Research Center, Taiwan, and is identical to the culture used by Pan et al. (2017). Two stock cultures of *A. royi* were kept in 100 l tanks. The copepods were cultivated in a 20 psu mixture of 0.2 µm UV filtrated seawater and demineralized water (culture water) at 25°C, with aeration and no light. Stock cultures were fed every second day with *I. galbana* and *R. salina*, reaching approximately 120,000 cells ml^−1^ and 20,000 cells ml^−1^, respectively.

### Copepod feeding regimes

Four different copepod feeding regimes were applied, one for each species of microalgae. Algae diet densities were calculated to correspond to the carbon content equal to 100,000 cells ml^−1^ of *I. galbana*, as referenced by Pan et al. (2017). The results of a prior experiment revealed the densities to equal 41,300, 24,500 and 14,000 cells ml^−1^ for *D. tertiolecta*, *R. salina* and *T. suecica*, respectively (see Table 3 for phytoplankton characteristics). The algae diets were administered *ad libitum* corresponding to 1140 µg C l^−1^, *sensu* Berggreen et al. (1988). Densities in algae batch cultures and copepod treatments were measured daily on a Beckman Coulter Multisizer 4e.

**Table 3.**
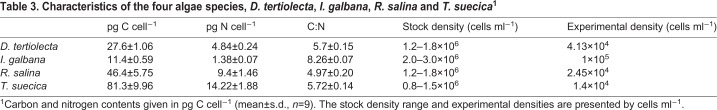
**Characteristics of the four algae species, *D. tertiolecta*, *I. galbana*, *R. salina* and *T. suecica*^1^**

### Feeding experiment

#### Experimental setup

*Apocyclops royi* nauplii were separated from the stock cultures by 125 µm and 53 µm mesh filters and captured in the latter. The nauplii were rinsed with freshly prepared culture water and separated into four 5 l tanks with a 16:8 h light cycle at the densities of approximately 3.25 ind ml^−1^, one for each algae diet. Culture water was changed every 4 days. The copepods were fed daily for 8 days at which point reproduction began. Adults and nauplii were separated by 250 µm and 53 µm mesh filters. The nauplii were placed in triplicate 800 ml beakers with fresh culture water at a density of approximately 0.33 ind ml^−1^. The copepods were fed daily for 8 days, and adults were separated by a 250 µm mesh filter. Adults from the second generation were placed in fresh culture water for 24 h immediately after separation. This was done to remove gut content to ensure pure copepod tissue for FA analysis.

For the FA analysis, triplicate samples were prepared with approximately 500 µg C for the algae and 20 individuals per sample for adult copepods. A preliminary experiment concluded this to be a sufficient number of adult copepods to get FA contents above detection limit. The algae and copepods were filtrated onto 25 mm Whatman GF/C filters and rinsed with MilliQ water. The filters were stored in 7.5 ml Pyrex vials and stored at −80°C for later FA analysis.

#### FA extraction

The samples were freeze-dried in a Christ-Alpha 1-2 (Osterode am Harz, Germany) equipped with a vacuum pump for 24 h. This was done to remove water and crush cell membranes. To the Pyrex vials 3 ml of 2:1 chloroform:methanol (v:v) was added according to Folch et al. (1957). An aliquot of 20 µg C23:0 FA methyl ester (FAME) was added as an internal standard. The vials were then stored for 24 h at −20°C for extraction. The following procedure for transesterification and preparation of samples was based on Drillet et al. (2006). Approximately 1.7 ml of the solutions was transferred to GC vials. The chloroform:methanol solution was evaporated by placing the GC vials on a heating block at 60°C under a stream of nitrogen. To the dry lipids 1000 µl of methanol/toluene/acetyl chloride (85:66:15) solution was added. The GC-vials were capped and left at 95°C for 2 h for transesterification. Next, 500 µl 5% NaHCO_3_ was added to remove excess acid from the organic phase. The solution was mixed and let to settle. The organic phase was washed twice with heptane and transferred to a new GC-vial. The solution was dried on a heating block at 60°C under a stream of nitrogen, and 500 µl chloroform was added. The samples were analyzed on Agilent GC 6890 N (Wiesental, Waghäusel, Germany) with an Agilent J&W DB-23 column (60 m×250 µm×0.25 µm) with helium as carrier gas. Initial temperature was 50°C and increased in a rate of 25°C min^−1^ until 200°C was reached, where it was held for 10 min. Hereafter the temperature increased at a rate of 5°C min^−1^ until 250°C, where it was held for 3 min. Standard calibration curves were created using FAME in varying concentrations while keeping the internal standard C23:0 constant. The samples were analyzed in MSD Chemstation E.02.02.1431, Agilent Technologies, by monitoring the specific ions: 55, 74, 79 and 81.

### Transcriptome and gene expression analysis

#### Experimental setup

Two separate cultures of *A. royi* were fed the microalgae *I. galbana* and *D. tertiolecta*, respectively. These two algae species were chosen as *I. galbana* contains large amounts of DHA and *D. tertiolecta* does not contain EPA and DHA, therefore mimicking environments with high- and low-PUFA seston. The cultures were kept at 25°C, 20 psu in dark conditions and fed daily. The cultures were managed for 2 months, equal to ∼7–8 generations, prior to sampling.

The copepods were starved in clean 0.2 µm UV filtrated 32 psu seawater for 2 h to empty their guts prior to collection to minimize contamination. All life stages of *A. royi* from each culture were caught on a 53 µm filter. Four analytical replicates were prepared for each of the two feeding regimes. Each replicate consisted of hundreds to thousands of individuals. The copepods were flushed with fresh 0.2 µm UV filtrated seawater up to four times by successive cold-sedimentation, where concentrated animals in 50 ml tubes were put on ice for 15 min to sediment, after which the top 45 ml seawater was removed, and the copepods were resuspended in fresh, 0°C precooled 0.2 µm UV filtrated seawater. Each sample was then inspected in a petri dish under a dissecting microscope and any remaining large lumps of algae mass or other non-copepod material was removed. Samples were sedimented again in 1.5 ml Eppendorf tubes and any remaining water removed with a small tip Pasteur pipettor. RNAlater was added to the copepods in a portion of 200 µl for those fed *I. galbana* and 500 µl for those fed *D. tertiolecta* ensuring a factor of at least 1:10 of copepods in RNAlater. Samples was kept in a fridge for 24 h and frozen at −20°C until use.

#### RNA extraction and sequencing library construction

RNA was extracted with RNeasy (Qiagen) according to protocol. Prior to extraction, residual RNAlater was removed and the animals were ground in 20 µl buffer RTL with a 1.5 ml RNase-Free Pellet Pestle (Kimble Chase) mounted on a Kontes Pellet Pestle motor (Kimble Chase) for 1 min on ice, before adding the remaining volume of Buffer RTL (330 µl).

A sequencing library for each of the eight samples was immediately prepared from 1 µg total RNA using the Truseq stranded mRNA protocol (Illumina) and pooled equimolarly using a KAPA qPCR system (Roche) and a Bioanalyzer 2100 (Agilent Biotechnology). The samples were sequenced on a NextSeq500 (Illumina) using a 1×150 bp ‘mid’ kit.

#### Data handling and analysis

Basic statistics and data handling were done in a UNIX environment using Biopieces (Hansen, MA, www.biopieces.org, unpublished). All data has been deposited in the EBI database under the project accession PRJEB28764. Adapters and low-quality bases were trimmed with Adapterremoval v. 2.0 (Schubert et al., 2016) with the following switches: –trimns –trimqualities.

#### Gene annotation and identification of putative *FAD* or *ELO* genes

Putative genes belonging to the gene families of elongation of fatty acids (*ELO*) and fatty acid desaturase (*FAD*) were identified using a dual BLAST and Hidden Markov Model (HMM) approach. Briefly, all >75.000 *A. royi* GHAJ01 sequences were BLASTed [BLASTx v. 2.2.31+, max e-value 0.0001 (Altschul et al., 1997)] against the Pfam (Finn et al., 2016) seed database of representative sequenced for the families *ELO* (PF01151) and *FAD* (PF00487), downloaded 30 May 2018. In parallel, the HMM of the same Pfam family SEED databases were searched against all translated *A. royi* GHAJ01 transcripts using transeq (all six reading frames, EMBOSS:6.6.0.0) using hmmscan from the HMMer v. 3.1b1 (e-value<0.0001, http://hmmer.org/). All genes, from which an isoform was found by either BLAST or HMM to be *ELO* or *FAD* like, were annotated using BLAST2GO (B2G) v. 5.1 (Götz et al., 2008) and the standard workflow on the B2G cloud in June, 2018 (GOmapping v. 2018.04). Only *ELO* and *FAD* transcripts specifically annotated by the B2G pipeline as relevant for the n-3 PUFA synthesis were further analyzed. In order to obtain complete genes for potential fragmented desaturase sequences, trimmed reads from all eight replicates were mapped to the two read clusters DN_52334 (GHAJ01038077-9) and DN_55045 (GHAJ01039406-8) using Bowtie2 (Langmead and Salzberg, 2012) (parameters: –local). The reads were then extracted and reassembled using SPAdes3.12 (Bankevich et al., 2012) (parameters: –rna) to obtain complete desaturase genes, which were reverse-complemented before further analysis. See Fig. S1 for alignment between GHAJ01038077-9 and GHAJ01039406-8 and the identified complete genes. Desaturase genes in the existing 22 copepod transcriptome assemblies (TSA) were identified with the BLAST, HMM and B2G approach described above. From the copepod TSA putative and known desaturases, we selected 26 genes highly similar to GHAJ01038077-9 and GHAJ01039406-8 for alignment in CLCgenomics 11.0 (Qiagen) along with the putative Δ5 and Δ6 desaturase sequences used in Wu et al. (2018). A neighbor-joining tree was constructed from the amino acid sequences trimmed to the conserved regions (start: HPGG, end: Q/HXXHHLFP) using standard parameters and 1000 bootstrap replicates in CLCgenomics 11.0.

#### Differential expression analysis

Differential expression analysis was performed in CLC genomics 11.0 with the RNA-Seq Analysis workflow using one reference sequence per transcript (GHAJ01+complete *FAD* genes), with reverse strand specificity. The CLC rna-seq workflows use TMM normalizations, similarly to the normalization in EdgeR (Robinson et al., 2010). The differential expression for RNA-Seq workflow from CLCgenomics 11.0 was used to produce the statistical comparison of the replicates using default parameters and testing for differential expression due to feeding regime across all group pairs. A heat map showing Euclidian distances for all annotated *FAD* and *ELO* transcripts, including the complete *FAD* genes identified above, principal component analysis (PCA), and statistical analysis testing differential expression due to feeding regime comparing all group pairs were also created in CLC genomics 11.0.

### Statistics

All mean values in the text are presented with±s.d. Fatty acid content of algae and copepods were normalized as fractions (%) of total FA content measured. Significant differences in contents of FAs of interest (n-3 PUFA and the immediate precursors of ALA; C16:0, C18:0, C18:1 and C18:2) in the copepod treatments were tested. For gene expression analysis PCA was performed on the expression value sets to confirm the groupings of the samples. Significant differences in expression levels were tested between groupings.

Significance level for all tests was set at 0.05. Normality was tested with Shapiro-Wilk tests and equal variances with Brown-Forsythe tests. Comparisons of two means were tested with *t*-test if parametric, and Mann–Whitney *U*-test if non-parametric. Comparisons of three or more parametric means were tested with one-way ANOVA and Tukey tests as post-hoc tests. If non-parametric, datasets were tested with Kruskal–Wallis and Dunn's tests as post-hoc tests. All FA content tests were done in GraphPad Prism 7, while gene expression tests were done in CLC Genomics Workbench v. 11.0.

## Supplementary Material

Supplementary information
