# Supplementary material for: n-3 PUFA biosynthesis by the copepod Apocyclops royi documented using fatty acid profile analysis and gene expression analysis
Source: Biol Open. 2019 Feb 5;8(2):bio038331. doi: 10.1242/bio.038331 (PMC6398464; doi:10.1242/bio.038331)
Supplement: Supplementary information [file biolopen-8-038331-s1.pdf]

Supplementary information

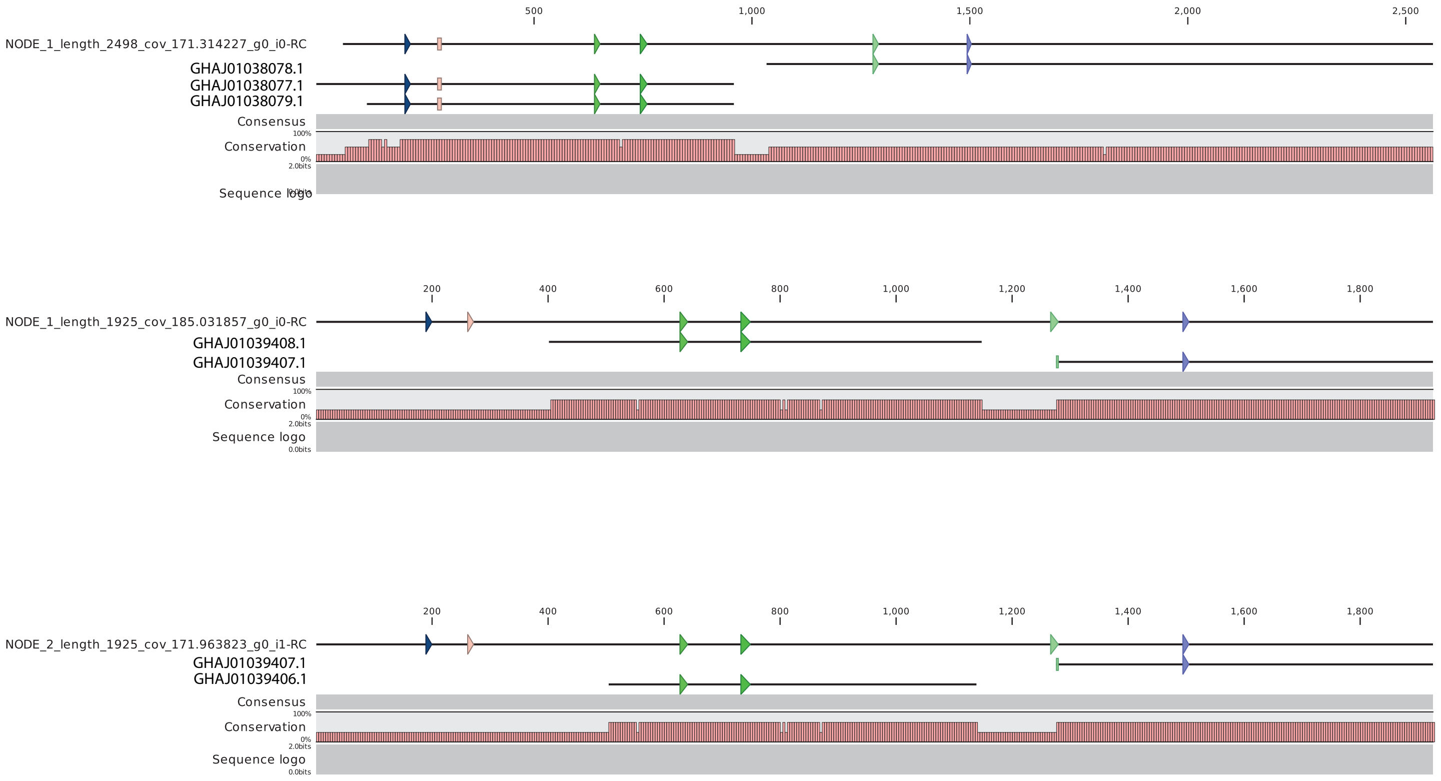

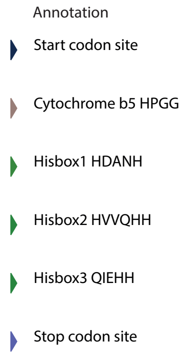

Figure S1. Aligning the nucleotide sequence of fragmented transcripts to complete genes. The three complete genes identified as putative front end desaturases were aligned to the initially identified fragmented genes from the GHAJ01 TSA assembly in CLCgenomics. For each com-plete gene, certain motifs were covered by the fragmented transcripts, but crucially, no fragment-ed gene covered all important front end desatu-rase motif. Notice that the fragmented isoforms and the complete genes do not share 100% sequence identity, likely because the assembly program faced with short reads with several SNPs had to chose a path through the variation. since Trinity was used for the initial assembly and SPAdes fro the complete gene assemble, the results are not identical.

Biology Open • Supplementary information

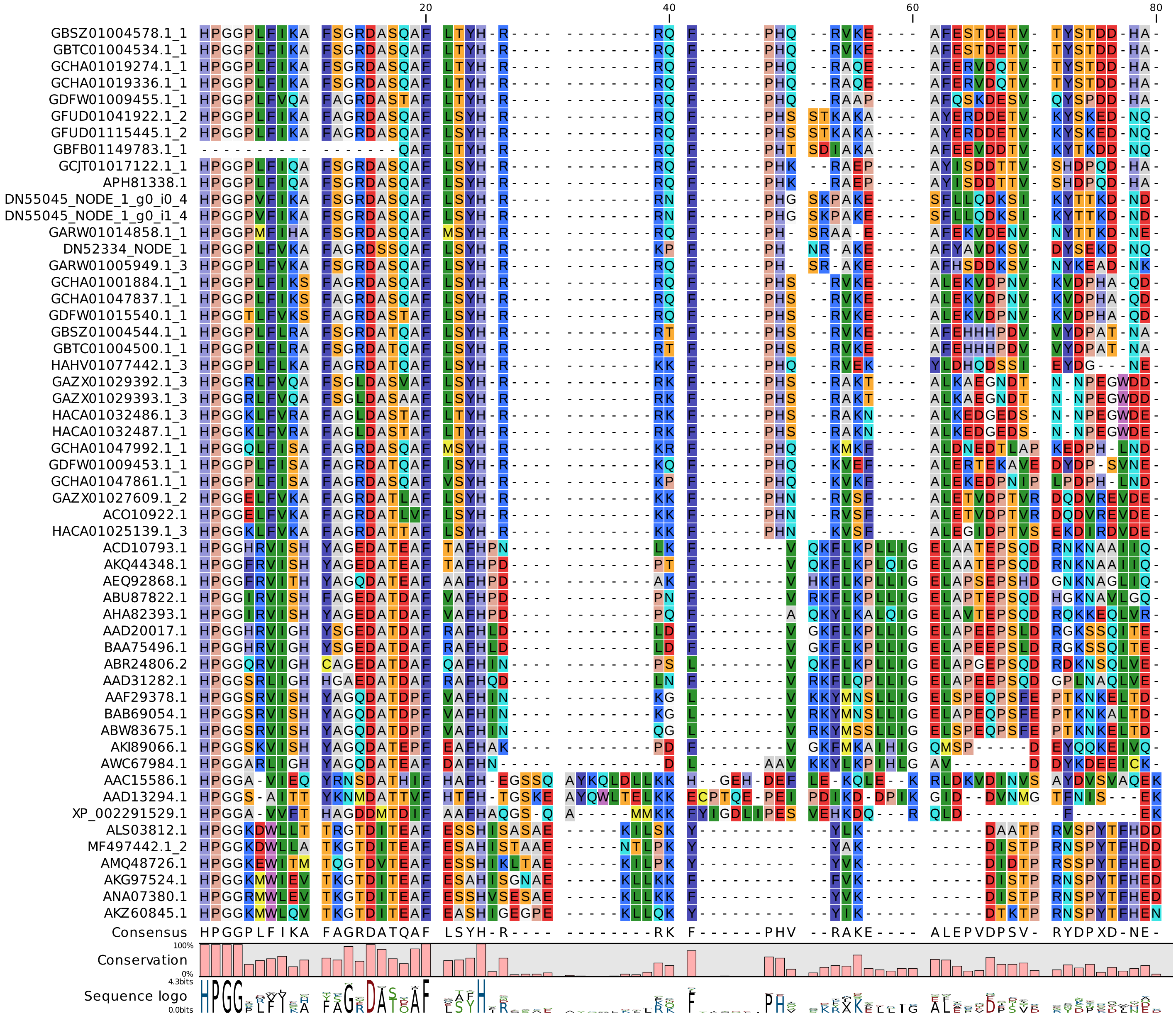

Biology Open • Supplementary information

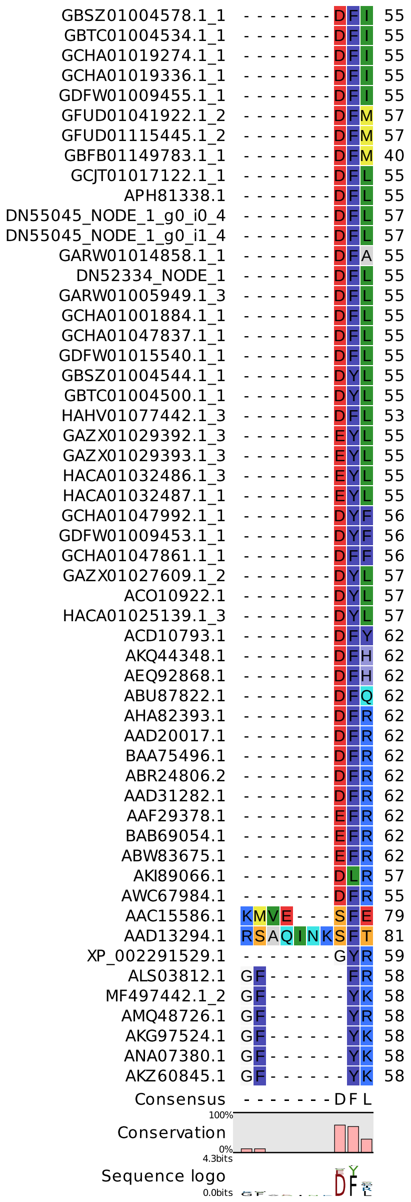

Biology Open • Supplementary information

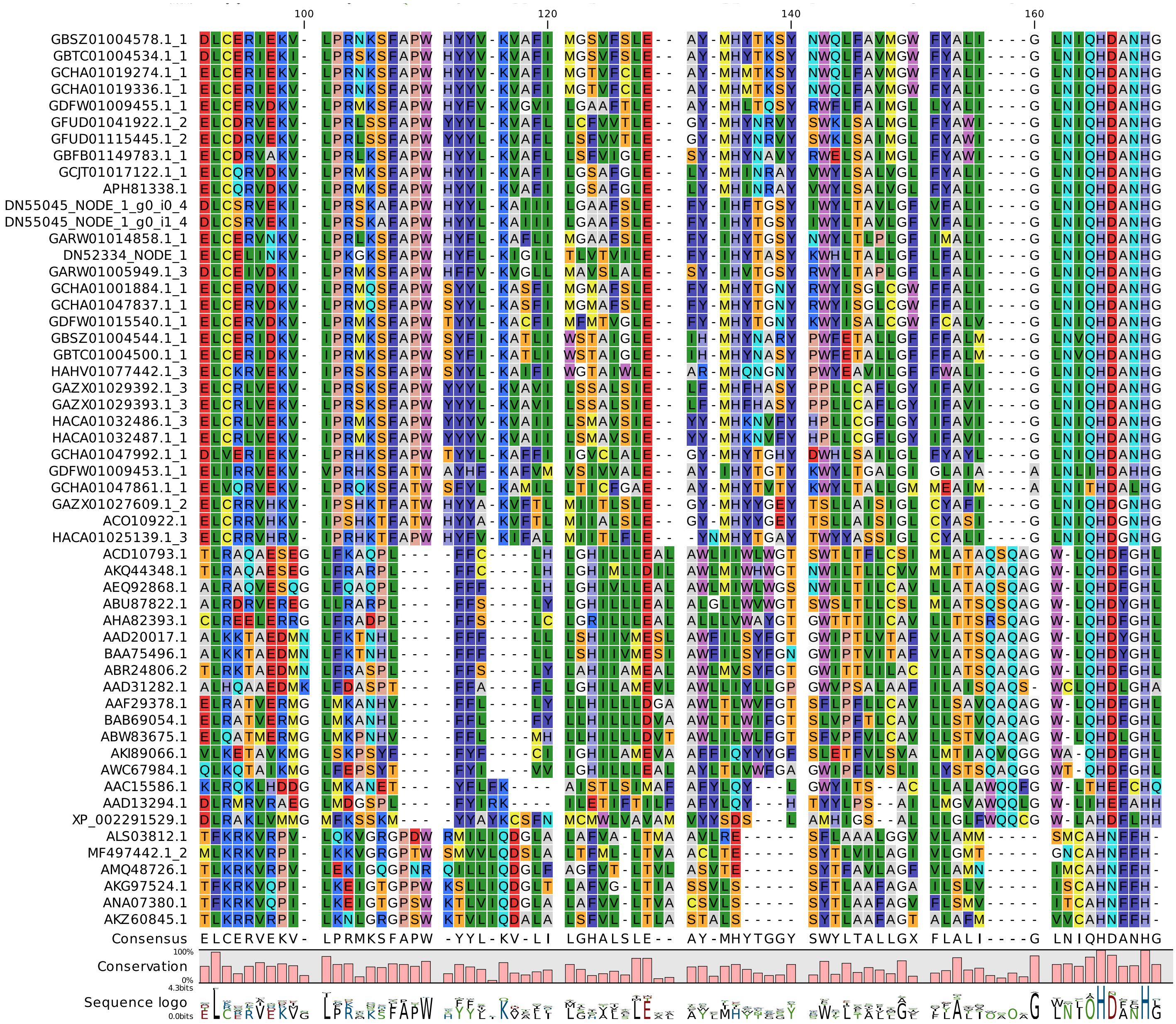
100
120
140
160
GBSZ01004578.1_1
GBTC01004534.1_1
GCHA01019274.1_1
GCHA01019336.1_1
GDFW01009455.1_1
GFUD01041922.1_2
GFUD01115445.1_2
GBFB01149783.1_1
GCJT01017122.1_1
APH81338.1
DN55045_NODE_1_g0_i0_4
DN55045_NODE_1_g0_i1_4
GARW01014858.1_1
DN52334_NODE_1
GARW01005949.1_3
GCHA01001884.1_1
GCHA01047837.1_1
GDFW01015540.1_1
GBSZ01004544.1_1
GBTC01004500.1_1
HAHV01077442.1_3
GAZX01029392.1_3
GAZX01029393.1_3
HACA01032486.1_3
HACA01032487.1_1
GCHA01047992.1_1
GDFW01009453.1_1
GCHA01047861.1_1
GAZX01027609.1_2
ACO10922.1
HACA01025139.1_3
ACD10793.1
AKQ44348.1
AEQ92868.1
ABU87822.1
AHA82393.1
AAD20017.1
BAA75496.1
ABR24806.2
AAD31282.1
AAF29378.1
BAB69054.1
ABW83675.1
AKI89066.1
AWC67984.1
AAC15586.1
AAD13294.1
XP_002291529.1
ALS03812.1
MF497442.1_2
AMQ48726.1
AKG97524.1
ANA07380.1
AKZ60845.1
Consensus
ELCERVEKV- LPRMKSFAPW -YYL-KV-LI LGHALSLE-- AY-MHYTGGY SWYLTALLGX FLALI----G LNIQHDANHG
Conservation
100%
0%
Sequence logo
4.3bits
0.0bits

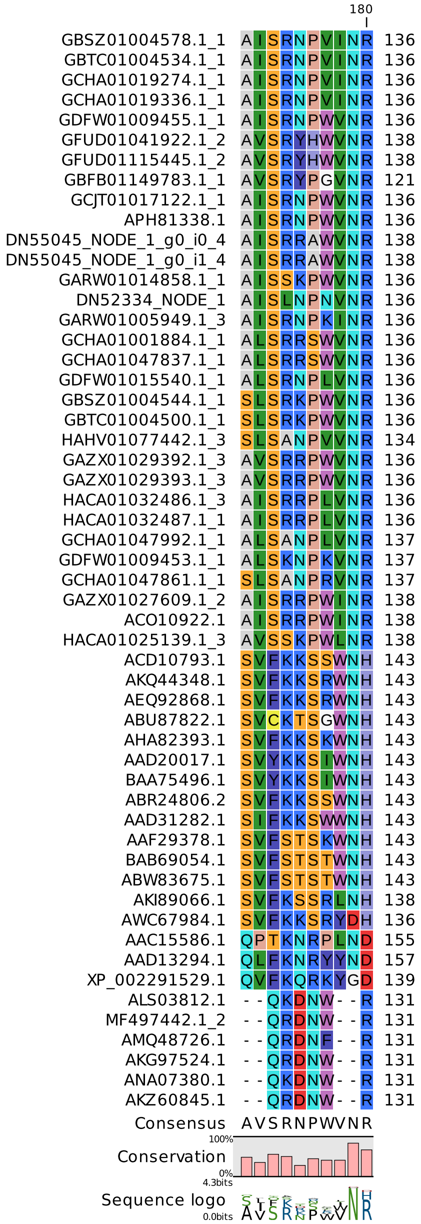
180
GBSZ01004578.1_1 AISRNPVINR 136
GBTC01004534.1_1 AISRNPVINR 136
GCHA01019274.1_1 AISRNPVINR 136
GCHA01019336.1_1 AISRNPVINR 136
GDFW01009455.1_1 AISRNPWVNR 136
GFUD01041922.1_2 AVSRYHWVNR 138
GFUD01115445.1_2 AVSRYHWVNR 138
GBFB01149783.1_1 AVSRYPGVNR 121
GCJT01017122.1_1 AISRNPWVNR 136
APH81338.1 AISRNPWVNR 136
DN55045_NODE_1_g0_i0_4 AISRRAWVNR 138
DN55045_NODE_1_g0_i1_4 AISRRAWVNR 138
GARW01014858.1_1 AISSKPWVNR 136
DN52334_NODE_1 AISLNPNVNR 136
GARW01005949.1_3 AISRNPKINR 136
GCHA01001884.1_1 ALSRRSWVNR 136
GCHA01047837.1_1 ALSRRSWVNR 136
GDFW01015540.1_1 ALSRNPLVNR 136
GBSZ01004544.1_1 SLSRKPWVNR 136
GBTC01004500.1_1 SLSRKPWVNR 136
HAHV01077442.1_3 SLSANPVVNR 134
GAZX01029392.1_3 AVSRRPWVNR 136
GAZX01029393.1_3 AVSRRPWVNR 136
HACA01032486.1_3 AISRRPLVNR 136
HACA01032487.1_1 AISRRPLVNR 136
GCHA01047992.1_1 ALSANPLVNR 137
GDFW01009453.1_1 ALSKNPKVNR 137
GCHA01047861.1_1 SLSANPRVNR 137
GAZX01027609.1_2 AISRRPWINR 138
ACO10922.1 AISRRPWINR 138
HACA01025139.1_3 AVSSKPWLNR 138
ACD10793.1 SVFKKSSWNH 143
AKQ44348.1 SVFKKSRWNH 143
AEQ92868.1 SVFKKSRWNH 143
ABU87822.1 SVCKTSGWNH 143
AHA82393.1 SVFKKSKWNH 143
AAD20017.1 SVYKKSIWNH 143
BAA75496.1 SVYKKSIWNH 143
ABR24806.2 SVFKKSSWNH 143
AAD31282.1 SIFKKSWWNH 143
AAF29378.1 SVFSTSKWNH 143
BAB69054.1 SVFSTSTWNH 143
ABW83675.1 SVFSTSTWNH 143
AKI89066.1 SVFKSSRLNH 138
AWC67984.1 SVFKKSRYDH 136
AAC15586.1 QPTKNRPLND 155
AAD13294.1 QLFKNRYYND 157
XP_002291529.1 QVFKQRKYGD 139
ALS03812.1 --QKDNW--R 131
MF497442.1_2 --QRDNW--R 131
AMQ48726.1 --QRDNF--R 131
AKG97524.1 --QRDNW--R 131
ANA07380.1 --QKDNW--R 131
AKZ60845.1 --QRDNW--R 131
Consensus AVSRNPWVNR
Conservation 100% 0%
Sequence logo 4.3bits 0.0bits

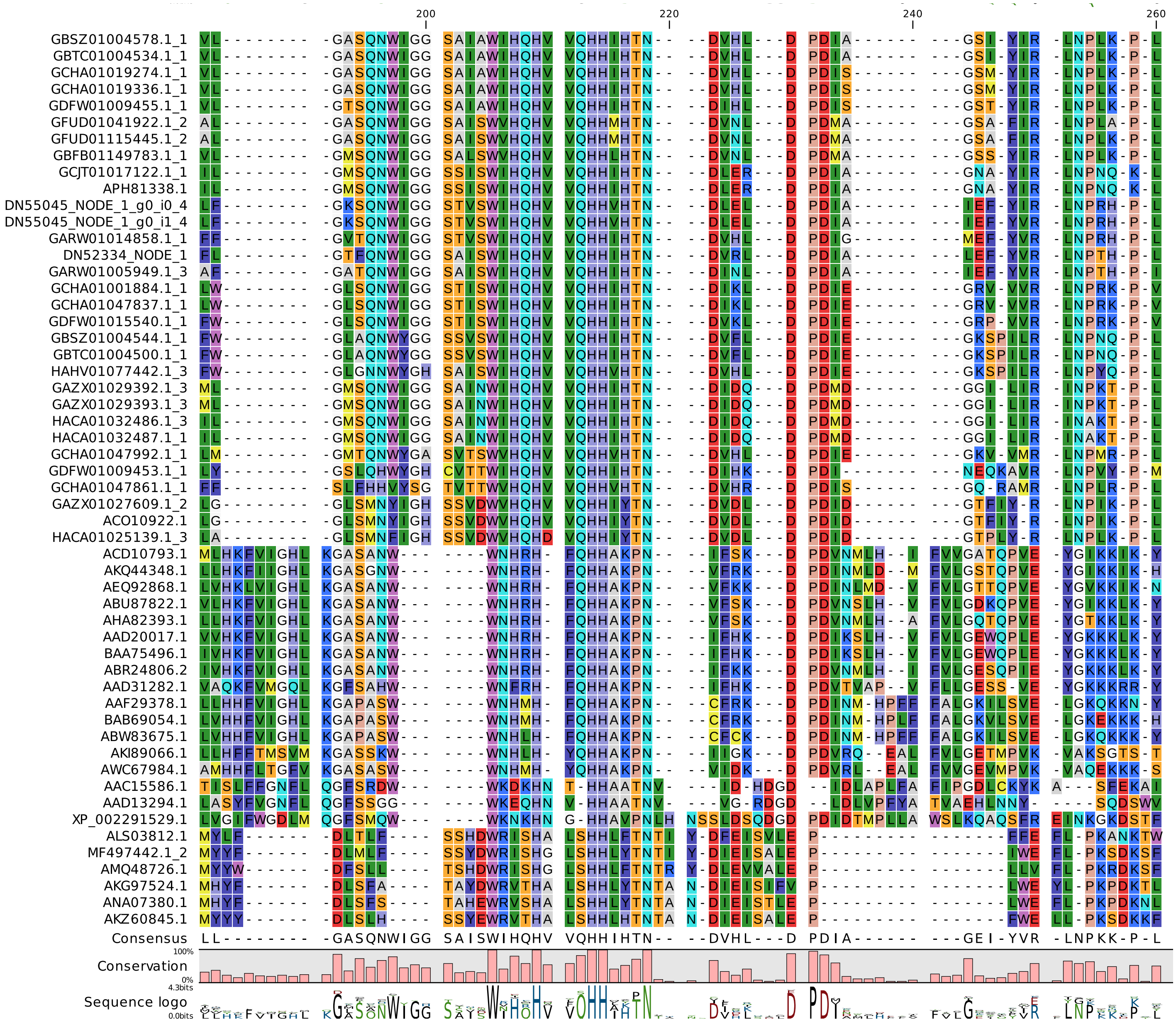
200
220
240
260
GBSZ01004578.1_1
GBTC01004534.1_1
GCHA01019274.1_1
GCHA01019336.1_1
GDFW01009455.1_1
GFUD01041922.1_2
GFUD01115445.1_2
GBFB01149783.1_1
GCJT01017122.1_1
APH81338.1
DN55045_NODE_1_g0_i0_4
DN55045_NODE_1_g0_i1_4
GARW01014858.1_1
DN52334_NODE_1
GARW01005949.1_3
GCHA01001884.1_1
GCHA01047837.1_1
GDFW01015540.1_1
GBSZ01004544.1_1
GBTC01004500.1_1
HAHV01077442.1_3
GAZX01029392.1_3
GAZX01029393.1_3
HACA01032486.1_3
HACA01032487.1_1
GCHA01047992.1_1
GDFW01009453.1_1
GCHA01047861.1_1
GAZX01027609.1_2
ACO10922.1
HACA01025139.1_3
ACD10793.1
AKQ44348.1
AEQ92868.1
ABU87822.1
AHA82393.1
AAD20017.1
BAA75496.1
ABR24806.2
AAD31282.1
AAF29378.1
BAB69054.1
ABW83675.1
AKI89066.1
AWC67984.1
AAC15586.1
AAD13294.1
XP_002291529.1
ALS03812.1
MF497442.1_2
AMQ48726.1
AKG97524.1
ANA07380.1
AKZ60845.1
Consensus LL-------- -GASQNWIGG SAISWIHQHV VQHHIHTN-- --DVHL---D PDIA------ ---GEI-YVR -LNPKK-P-L
Conservation
100%
0%
Sequence logo
4.3bits
0.0bits

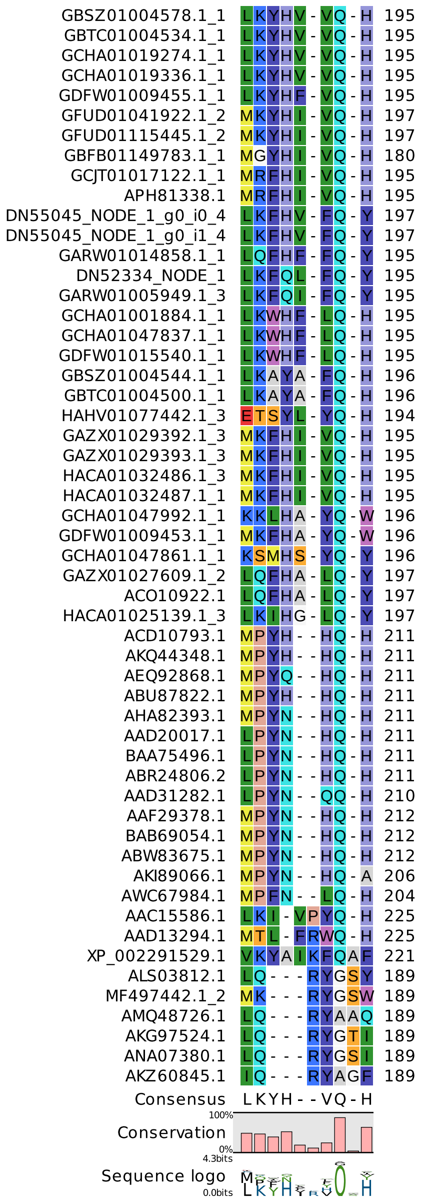
GBSZ01004578.1_1 LKYHV-VQ-H 195
GBTC01004534.1_1 LKYHV-VQ-H 195
GCHA01019274.1_1 LKYHV-VQ-H 195
GCHA01019336.1_1 LKYHV-VQ-H 195
GDFW01009455.1_1 LKYHF-VQ-H 195
GFUD01041922.1_2 MKYHI-VQ-H 197
GFUD01115445.1_2 MKYHI-VQ-H 197
GBFB01149783.1_1 MGYHI-VQ-H 180
GCJT01017122.1_1 MRFHI-VQ-H 195
APH81338.1 MRFHI-VQ-H 195
DN55045_NODE_1_g0_i0_4 LKFHV-FQ-Y 197
DN55045_NODE_1_g0_i1_4 LKFHV-FQ-Y 197
GARW01014858.1_1 LQFHF-FQ-Y 195
DN52334_NODE_1 LKFQL-FQ-Y 195
GARW01005949.1_3 LKFQI-FQ-Y 195
GCHA01001884.1_1 LKWHF-LQ-H 195
GCHA01047837.1_1 LKWHF-LQ-H 195
GDFW01015540.1_1 LKWHF-LQ-H 195
GBSZ01004544.1_1 LKAYA-FQ-H 196
GBTC01004500.1_1 LKAYA-FQ-H 196
HAHV01077442.1_3 ETSYL-YQ-H 194
GAZX01029392.1_3 MKFHI-VQ-H 195
GAZX01029393.1_3 MKFHI-VQ-H 195
HACA01032486.1_3 MKFHI-VQ-H 195
HACA01032487.1_1 MKFHI-VQ-H 195
GCHA01047992.1_1 KKLHA-YQ-W 196
GDFW01009453.1_1 MKFHA-YQ-W 196
GCHA01047861.1_1 KSMHS-YQ-Y 196
GAZX01027609.1_2 LQFHA-LQ-Y 197
ACO10922.1 LQFHA-LQ-Y 197
HACA01025139.1_3 LKIHG-LQ-Y 197
ACD10793.1 MPYH--HQ-H 211
AKQ44348.1 MPYH--HQ-H 211
AEQ92868.1 MPYQ--HQ-H 211
ABU87822.1 MPYH--HQ-H 211
AHA82393.1 MPYN--HQ-H 211
AAD20017.1 LPYN--HQ-H 211
BAA75496.1 LPYN--HQ-H 211
ABR24806.2 LPYN--HQ-H 211
AAD31282.1 LPYN--QQ-H 210
AAF29378.1 MPYN--HQ-H 212
BAB69054.1 MPYN--HQ-H 212
ABW83675.1 MPYN--HQ-H 212
AKI89066.1 MPYN--HQ-A 206
AWC67984.1 MPFN--LQ-H 204
AAC15586.1 LKI-VPYQ-H 225
AAD13294.1 MTL-FRWQ-H 225
XP_002291529.1 VKYAIKFQAF 221
ALS03812.1 LQ---RYGSY 189
MF497442.1_2 MK---RYGSW 189
AMQ48726.1 LQ---RYAAQ 189
AKG97524.1 LQ---RYGTI 189
ANA07380.1 LQ---RYGSI 189
AKZ60845.1 IQ---RYAGF 189
Consensus LKYH--VQ-H
Conservation
100%
0%
Sequence logo
4.3bits
0.0bits

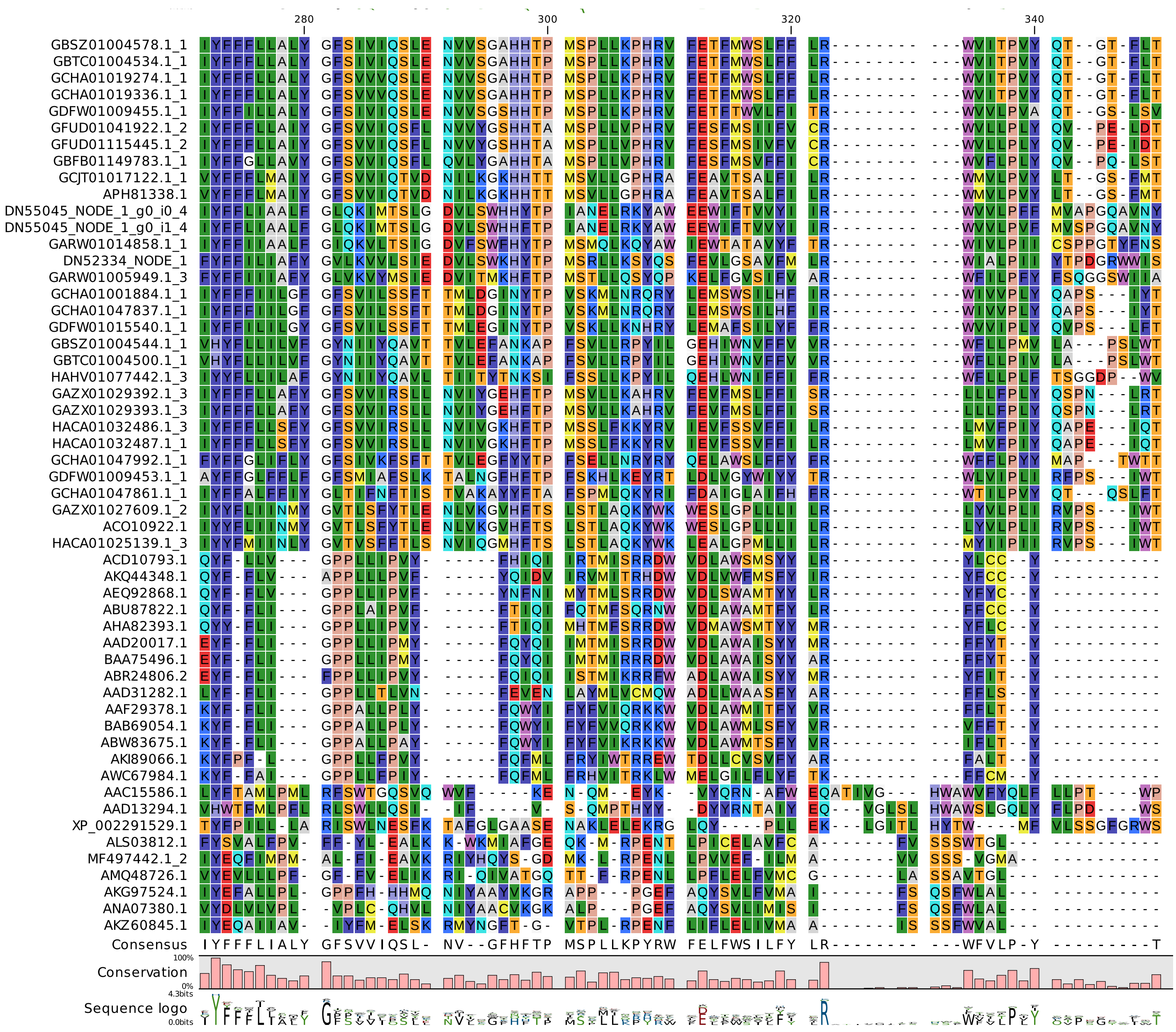

Biology Open • Supplementary information

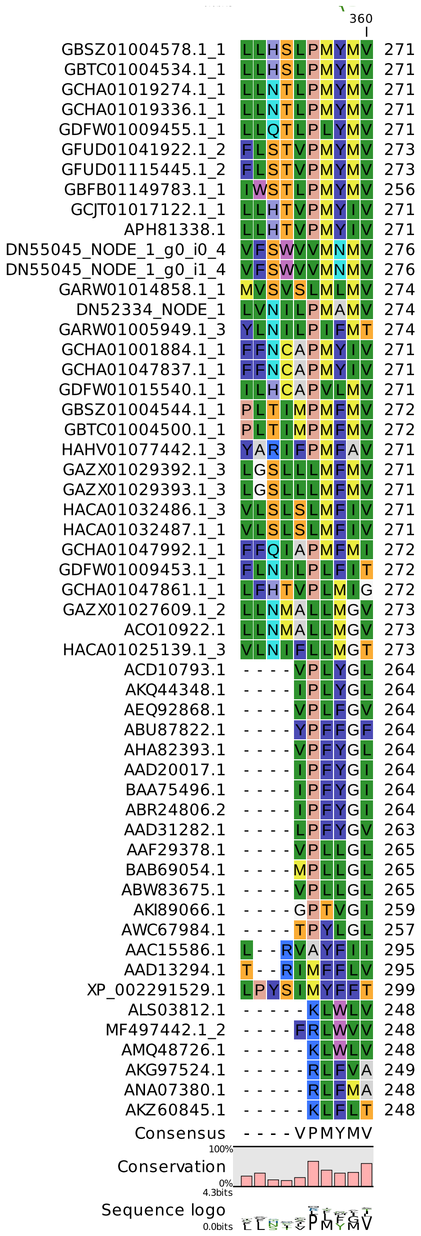
360
GBSZ01004578.1_1 LLHSLPMYMV 271
GBTC01004534.1_1 LLHSLPMYMV 271
GCHA01019274.1_1 LLNTLPMYMV 271
GCHA01019336.1_1 LLNTLPMYMV 271
GDFW01009455.1_1 LLQTLPLYMV 271
GFUD01041922.1_2 FLSTVPMYMV 273
GFUD01115445.1_2 FLSTVPMYMV 273
GBFB01149783.1_1 IWSTLPMYMV 256
GCJT01017122.1_1 LLHTVPMYIV 271
APH81338.1 LLHTVPMYIV 271
DN55045_NODE_1_g0_i0_4 VFSWVVMNMV 276
DN55045_NODE_1_g0_i1_4 VFSWVVMNMV 276
GARW01014858.1_1 MVSVSLMLMV 274
DN52334_NODE_1 LVNILPMAMV 274
GARW01005949.1_3 YLNILPIFMT 274
GCHA01001884.1_1 FFNCAPMYIV 271
GCHA01047837.1_1 FFNCAPMYIV 271
GDFW01015540.1_1 ILHCAPVLMV 271
GBSZ01004544.1_1 PLTIMPMFMV 272
GBTC01004500.1_1 PLTIMPMFMV 272
HAHV01077442.1_3 YARIFPMFAV 271
GAZX01029392.1_3 LGSLLLMFMV 271
GAZX01029393.1_3 LGSLLLMFMV 271
HACA01032486.1_3 VLSLSLMFIV 271
HACA01032487.1_1 VLSLSLMFIV 271
GCHA01047992.1_1 FFQIAPMFMI 272
GDFW01009453.1_1 FLNILPLFIT 272
GCHA01047861.1_1 LFHTVPLMIG 272
GAZX01027609.1_2 LLNMALLMGV 273
ACO10922.1 LLNMALLMGV 273
HACA01025139.1_3 VLNIFLLMGT 273
ACD10793.1 ----VPLYGL 264
AKQ44348.1 ----IPLYGL 264
AEQ92868.1 ----VPLFGV 264
ABU87822.1 ----YPFFGF 264
AHA82393.1 ----VPFYGL 264
AAD20017.1 ----IPFYGI 264
BAA75496.1 ----IPFYGI 264
ABR24806.2 ----IPFYGI 264
AAD31282.1 ----LPFYGV 263
AAF29378.1 ----VPLLGL 265
BAB69054.1 ----MPLLGL 265
ABW83675.1 ----VPLLGL 265
AKI89066.1 ----GPTVGI 259
AWC67984.1 ----TPYLGL 257
AAC15586.1 L--RVAYFII 295
AAD13294.1 T--RIMFFLV 295
XP_002291529.1 LPYSIMYFFT 299
ALS03812.1 -----KLWLV 248
MF497442.1_2 ----FRLWVV 248
AMQ48726.1 -----KLWLV 248
AKG97524.1 -----RLFVA 249
ANA07380.1 -----RLFMA 248
AKZ60845.1 -----KLFLT 248
Consensus ----VPMYMV
Conservation
100%
0%
4.3bits
Sequence logo
0.0bits

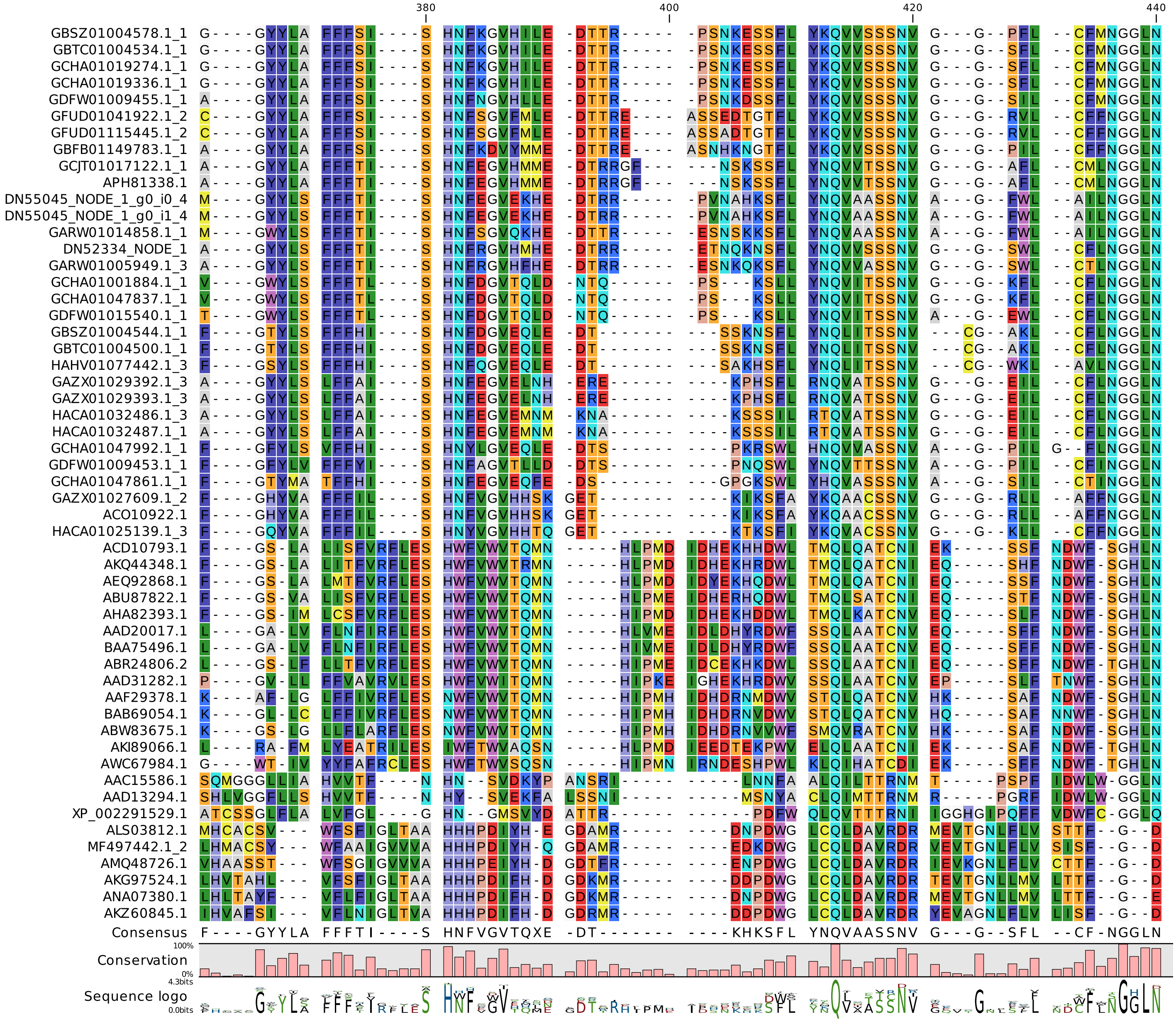
380
400
420
440
GBSZ01004578.1_1
GBTC01004534.1_1
GCHA01019274.1_1
GCHA01019336.1_1
GDFW01009455.1_1
GFUD01041922.1_2
GFUD01115445.1_2
GBFB01149783.1_1
GCJT01017122.1_1
APH81338.1
DN55045_NODE_1_g0_i0_4
DN55045_NODE_1_g0_i1_4
GARW01014858.1_1
DN52334_NODE_1
GARW01005949.1_3
GCHA01001884.1_1
GCHA01047837.1_1
GDFW01015540.1_1
GBSZ01004544.1_1
GBTC01004500.1_1
HAHV01077442.1_3
GAZX01029392.1_3
GAZX01029393.1_3
HACA01032486.1_3
HACA01032487.1_1
GCHA01047992.1_1
GDFW01009453.1_1
GCHA01047861.1_1
GAZX01027609.1_2
ACO10922.1
HACA01025139.1_3
ACD10793.1
AKQ44348.1
AEQ92868.1
ABU87822.1
AHA82393.1
AAD20017.1
BAA75496.1
ABR24806.2
AAD31282.1
AAF29378.1
BAB69054.1
ABW83675.1
AKI89066.1
AWC67984.1
AAC15586.1
AAD13294.1
XP_002291529.1
ALS03812.1
MF497442.1_2
AMQ48726.1
AKG97524.1
ANA07380.1
AKZ60845.1
Consensus
Conservation
100%
0%
Sequence logo
4.3bits
0.0bits

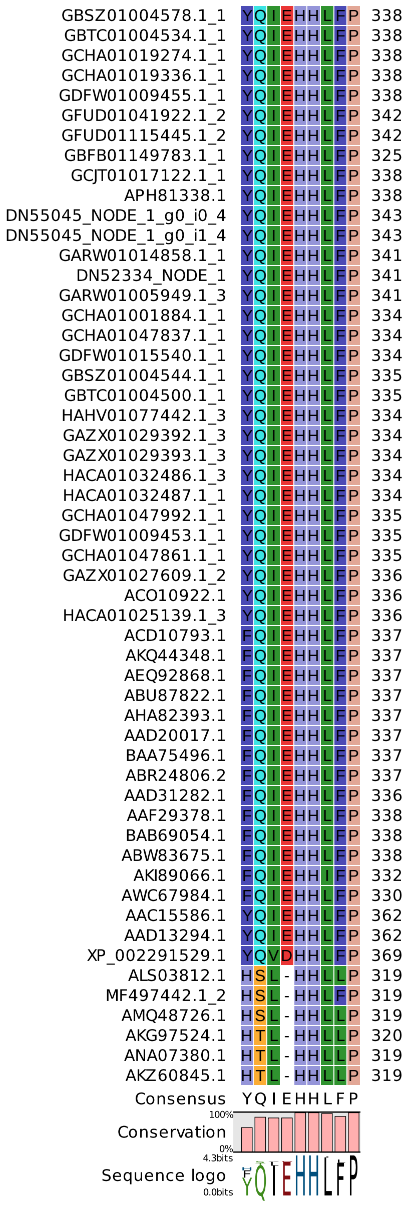

Figure S2. txt overview of identified copepod desaturase genes including AA sequence.

Biology Open • Supplementary information

Table S1. Fasta nt sequence of differently expressed desaturase genes.

>NODE_1_length_2498_cov_171.314227_g0_i0

GGGCCTTTTTTGCCGGTATGGTATGACAGTACGGCAGCAAAGTACAGTAGAATGGACGAC

CAGATTCTGGGGACAAAATTATTTGGCACAATTGTACTACTTTACCGTACCCGTCTGTCG

TACCAGGGTTGTACTTTTAAACGCTCCATATTTCTGATGCCGAGAACTCGAATAAATACA

CAGTAAAAAAAACATTTAATTGTCTTACATTGCATTGGATTACTGCAATAAATTGTACTA

ATGAGACATAATAGAAAAAATTAATTCTAAAAAGAAAATTGAAGTCAACTGCGCAAGACT

TGCCATTTTAATCAATATCCTTCTATTCTATTCAATAACTAAGAAATTATTGATGTTGAC

TAAAAATAGTTGAATATTTCAGATTTTGTTTCTCTTAAAAGTATTTTTTTCGAAAATTCG

AGACTATCCTCTCAAGCCGCAGAAGATTCTTTATAGTTTATGAGAACATATTTTTGATTG

TAATAATTAATGATGCTTCAATCATTCATGATAAAAAATTTCCACCTAACATCATCCATG

ATTTCGCAAAGTTTTTTTAATGATGCACAAAATTCGCTTCCTTGTCGCTACTCCCAAACT

GTGGCGGTCATATTTAAAATTGTCGATTCAGCACTTAACCCTGCCAGGGTCATCGAAATC

CCTATTGCGGCAATGGGTTGACGTCGTAATTCTCAAAAGAGCGTTGCTCAAATAAAGTAC

GCCTCAATAGAGCACCATCTAAACAATAGTGCATTTGGGCAGTGACAGTGGGCAGGGTCC

ATTTAAAAGGTGATATTTGATTTTAAACAAGACAAGTGCACTATTTTTCAGAACGCCACC

TCTTCAGAGTAGTTGAATATATACGTACACATACTTGAATTCTTTTCAGGAAGCTGTGTT

TGTTTTTGAACTCGTATTGTATGTTATTTGGCAACGTAGCAATGTAATCTTAAAAAATCT

TTTTTCTTTCATCAATATGTTTTCTCATTCTCAGGTGGAGGGGATTGCACGTCGGGTGGG

GTGGGGTGTTGCAGGGTCGAAGGGCATTATGTGGCTTATGAAACAATGGGCATTTTAGTT

GCCGCCTTCTCCATAACAACCGAATTCGGCATCTCACTTTCTCCCATGTCCATGAGGTGC

TTGATGCACGCACGAAGGTTTGCGTCGATGGTTGTGAAATGGACGTAGGGGATCTTGCGC

TCATCGCAAAATTTCTTAACGTGTGGTGCGATTGTCGGATAATGTGTGTGATTTATCCGA

GGAAAAAGATGATGTTCGATCTGGTAATTGAGACCTCCATTTAGAAAACAAAGCCACGAA

CCACCGACGTTTGAGGAAGAAACAACCTGATTGTAGAGGAAGGAGTTCTTTTGGTTCGTT

Biology Open • Supplementary information

TCTCTTCTAGTGTCCTCGTGCATGTGGACACCTCTAAAGTTGTGGCTGATTGTGAAGAAG

AAAGACAGGTAATAGCCGGCCACCATAGCCATAGGCAGTATGTTCACTAAGGAGATCCAC

CACCTTCCGTCCGGCGTGTAGATAATGGGAAGGGCGATCCATCTAAGCATGAAGACTGCT

GAGCCCAAGACTTCAAAGGATTGGTAGCTTTTAAGAAGACGCGACATCGGAGTGTAATGT

TTCCAAGAAAGGACATCCTCAATGGATAGAACAACCTTGAGGACGCCGTAAAACGCGATA

AGGATGAAAAAATAGAAGTACTGGAATAGTTGAAACTTGAGCAGAGGGTGCGTCGGATTC

AATCTCACGTAAAACTCGAGAGCAATATCCGGATCGAGCCTGACATCGTTCGTGTGGATG

TGATGCTGGACAACATGTTGATGGATCCATGAAATGGCACTGCCACCGATCCAGTTCTGG

AAGGTTCCAAGAAATCGATTCACATTCGGATTCAAGGAAATTGCTCCGTGATTTGCGTCG

TGCTGGATGTTCAAACCGATGAGAGCAAATAGGAATCCCAACAAAGCTGTTAGATGCCAT

TTGTAGGATGCAGTGTAGTGGATGTAAAATTCCAAAATGACTGTCACCAAGGTTAAGATT

CCGATCTTAAGAAAGTAGTGCCACGGAGCAAACGACTTCCCCTTTGGTAAAACCTTATTA

ATGAGTTCGCATAGTTCGAGGAAGTCTTGATTATCCTTTTCGCTGTAGTCGACAGATTTA

TCGACAGCATAAAAAGCTTCTTTGGCTCGATTGTGTGGAAAAGGTTTGCGATGATACGAC

AAAAATGCTTGAGATGAATCACGGCCGGCAAATGCCTTGACGAATAAAGGTCCACCGGGA

TGAATTTTAGCCAGTTGCTGAGCAGAATAAAGCTTGTCTTCTACTCGAATCAATTCATCA

GGAACGCGGCTCGGCATTGTGAGATGAAATATCTAAATAGTTGATAGAATGAAAGTAAAT

GAACTATTCCGGTGTTACCGATGCTTTCTTCTGGTACATTTATATACAAAATTTAAAGTT

TGCGCTGTGTTGTTTTTGGAAACTGGTTAAAAAGTTTC

>NODE_1_length_1925_cov_185.031857_g0_i0

CGCCGCCAAAACAACTTTTAGTGATGGAGAAAAATTTTTAGAAAAATGAGTAAAATAACT

ATACCTCCTTAGTTTTTTAGATGACCTGAGAACTATTTCTCATTTTAAAATTGTTCCAAG

TTAACTTTTAATGATTAAAATGAGATCATTGACGGCATCTCTTTAATAGGTCTAAGTCAA

TCAAACATTTATGTAAAAAAAATGCGTGATACTTTAAAAGACATATATTTCTGCATTGAT

Biology Open • Supplementary information

ATTCGAACTCTGCTAAAAGAGCAATATGCATTTAATCTGTTAACAAGGTGAAATTTTCAT

TTCTATACAAGGGATACTAATTCTAAATTTGTATCTTGTGATTGATTCGGTTTTCGTTGC

CACGTCAAATGACGCTTTCTGTCTGTTTTATAATTCCACAAACCATAAGATTTTGGGAGT

TTAAGAAACGATATGCATTTTTGCAGAAACTTTTTCGGAGGGGTTGACGAAGCCTTCGAT

GTTGAAGTTCTTGTTGCTGCCCATTTCCGCCAGATGTTGCACCAAGGCTCTCACGTTTTG

TGGAACGGACTCGAAGTGCGTGTACGGAATCTTTCGCTCCTCGCAGAACTGCCTCACTAC

GGGCGCGATGTAATAGTAGTGAGTGTGATTGATCCTCGGGAACAGGTGGTGTTCGATTTG

GTAGTTGAGGCCGCCGTTCAGGATTGCCAGCCAGAATCCAGCGACATTGGATGAAGCGGC

AACCTGGTTGTATAGGAAAGACTTGTGTGCGTTGACTGGTCTTCTCGTGTCTTCATGCTT

TTCGACACCTTCAAAGTTGTGGCTGATCGTGAAGAAGAAAGACAGGTAGTAGCCCATCAC

CATGTTCATCACCACCCAGCTGAAGACGTAGTTAACGGCCTGCCCAGGTGCAACCATGAA

GAAGGGCAAGACGACCCACCTGATGATGTAGACAACGGTGAAAATCCATTCCTCCCAAGC

GTACTTACGTAACTCGTTGGCGATCGGAGTATAGTGGTGCCAACTGAGGACATCGCCGAG

ACTCGTCATGATTTTTTGCAATCCGAAAAGAGCAGCAATGAGGAAGAAATAAATGTACTG

AAAGACATGAAACTTCAGCAGAGGATGTCTAGGGTTCAGTCGAATGTAAAACTCGATGGC

AATGTCGGGATCGAGCTCGAGATCGTTGGTGTGAACGTGGTGTTGAACGACATGTTGGTG

AATCCATGAGACAGTGCTACCCCCGATCCAATTTTGAGATTTTCCAAAGAGTCGATTGAC

CCATGCCCTTCTCGAAATTGCGCCATGGTTGGCATCGTGCTGAATATTTAAACCAATTAA

AGCGAAGACGAACCCAAGGACTGCAGTCAGATACCAAATGTAGGAGCCGGTGAAGTGGAT

GTAAAACTCGAGTGAGAATGCAGCTCCAAGGATGATGATCGCCTTCAGGTAGTAGTGCCA

CGGAGCAAAAGCCTTTGACCGCGGAAGTATCTTCTCGACTCTACTGCATAGGTCCAAAAA

GTCATCGTTGTCCTTTGTGGTGTACTTAATACTTTTGTCTTGTAAGAGGAAAGATTCTTT

TGCAGGCTTGCTGCCATGAGGAAAATTTCTTCTGTGGTAGGAAAGGAATGCCTGAGAGGC

GTCTCGACCCGAAAATGCCTTAATGAAGACCGGTCCACCTGGGTGAAGCTTGGACAACTT

Biology Open • Supplementary information

CTCAGCGGAGTAGACTTTGTCCTCTATTCGGATCTGACCCTCGGGGACTTCAGACATGTT

AATACAATCTTTTCTTATCCTGAAGCCCTGAACTCCGAGAAAACTGTTATTCTGATTTTC

TGACTTGGAACTTTGCGGCAGACAGATTTTCTCAGACAGCGGCAATGCAAAAATCGCACG

ATCGGCGAGAACTGTTTAAAATACTTTAAAGAACACAAAAAGCTCGATCATTTGCAGTAA

AAAAT

>NODE_2_length_1925_cov_171.963823_g0_i1

CGCCGCCAAAACAACTTTTAGTGATGGAGAAAAATTTTTAGAAAAATGAGTAAAATAACT

ATACCTCCTTAGTTTTTTAGATGACCTGAGAACTATTTCTCATTTTAAAATTGTTCCAAG

TTAACTTTTAATGATTAAAATGAGATCATTGACGGCATCTCTTTAATAGGTCTAAGTCAA

TCAAACATTTATGTAAAAAAAATGCGTGATACTTTAAAAGACATATATTTCTGCATTGAT

ATTCGAACTCTGCTAAAAGAGCAATATGCATTTAATCTGTTAACAAGGTGAAATTTTCAT

TTCTATACAAGGGATACTAATTCTAAATTTGTATCTTGTGATTGATTCGGTTTTCGTTGC

CACGTCAAATGACGCTTTCTGTCTGTTTTATAATTCCACAAACCATAAGATTTTGGGAGT

TTAAGAAACGATATGCATTTTTGCAGAAACTTTTTCGGAGGGGTTGACGAAGCCTTCGAT

GTTGAAGTTCTTGTTGCTGCCCATTTCCGCCAGATGTTGCACCAAGGCTCTCACGTTTTG

TGGAACGGACTCGAAGTGCGTGTACGGAATCTTTCGCTCCTCGCAGAACTGCCTCACTAC

GGGCGCGATGTAATAGTAGTGAGTGTGATTGATCCTCGGGAACAGGTGGTGTTCGATTTG

GTAGTTGAGGCCGCCGTTCAGGATTGCCAGCCAGAATCCAGCGACATTGGATGAAGCGGC

AACCTGGTTGTATAGGAAAGACTTGTGTGCGTTGACTGGTCTTCTCGTGTCTTCATGCTT

TTCGACACCTTCAAAGTTGTGGCTGATCGTGAAGAAGAAAGACAGGTAGTAGCCCATCAC

CATGTTCATCACCACCCAGCTGAAGACGTAGTTAACGGCCTGCCCAGGTGAAACCATGAA

GACGGGCAAGACGACCCACCTGATGATGTAGACAACGGTGAAAATCCATTCCTCCCAGGC

GTACTTACGTAGCTCGTTGGCGATCGGGGTAAAGTGGTGCCAACTGAGGACATCGCCGAG

ACTCGTCATGATTTTTTGTAATCCGAAAAGAGCAGCGATGAGGAAAAAATAAATGTACTG

Biology Open • Supplementary information

AAAGACATGAAACTTCAGCAGCGGATGTCTAGGGTTGAGTCGGACGTAAAACTCGATGGC

AATGTCCGGATCGAGCTCAAGATCGTTGGTGTGAACGTGGTGTTGAACGACATGTTGGTG

AATCCATGAGACAGTGCTTCCTCCGATCCAATTTTGAGATTTTCCAAAGAGTCGATTGAC

CCATGCCCTTCTCGATATTGCGCCATGGTTGGCATCGTGCTGAATATTTAAACCAATTAA

AGCGAAGACAAACCCAAGGACTGCAGTCAGGTACCAAATGTAGGAGCCGGTGTAGTGGAT

GTAAAACTCGAGTGAGAATGCAGCTCCAAGGATGATGATCGCCTTCAGGTAGTAGTGCCA

CGGAGCAAAAGCCTTTGACCGCGGAAGTATCTTCTCGACTCTACTGCATAGGTCCAAAAA

GTCATCGTTGTCCTTTGTGGTGTACTTAATACTTTTGTCTTGTAAGAGGAAAGATTCTTT

TGCAGGCTTGCTGCCATGAGGAAAATTTCTTCTGTGGTAGGAAAGGAATGCCTGAGAGGC

GTCTCGACCCGAAAATGCCTTAATGAAGACCGGTCCACCTGGGTGAAGCTTGGACAACTT

CTCAGCGGAGTAGACTTTGTCCTCTATTCGGATCTGACCCTCGGGGACTTCAGACATGTT

AATACAATCTTTTCTTATCCTGAAGCCCTGAACTCCGAGAAAACTGTTATTCTGATTTTC

TGACTTGGAACTTTGCGGCAGACAGATTTTCTCAGACAGCGGCAATGCAAAAATCGCACG

ATCGGCGAGAACTGTTTAAAATACTTTAAAGAACACAAAAAGCTCGATCATTTGCAGTAA

AAAAT

Table S2. Datasets used for FA desaturase and elongase identification.

Accession number	Data type	Species
GHAJ01	TSA	*Apocyclops royi*
GFWY01	TSA	*Acartia tonsa*
HAGX01	TSA	*Acartia tonsa*
GAXK01	TSA	*Calanus finmarchicus*
GBFB01	TSA	*Calanus finmarchicus*
GBXU01	TSA	*Calanus finmarchicus*
GBXT01	TSA	*Calanus glacialis*
HACJ01	TSA	*Calanus glacialis*
GAZX01	TSA	*Caligus rogercresseyi*
GFUA01	TSA	*Epischura baikalensis*
GARW01	TSA	*Eucyclops serrulatus*
GBGO01	TSA	*Eurytemora affinis*
GEAN01	TSA	*Eurytemora affinis*
GFWO01	TSA	*Labidocera madurae*
HACA01	TSA	*Lepeophtheirus salmonis*
GFUD01	TSA	*Neocalanus flemingeri*
GCJT01	TSA	*Paracyclopina nana*
GFCI01	TSA	*Pleuromamma xiphias*
GBSZ01	TSA	*Tigriopus californicus*
GBTC01	TSA	*Tigriopus californicus*
GCHA01	TSA	*Tigriopus japonicus*
GDFW01	TSA	*Tigriopus sp.*
HAHV01	TSA	*Tisbe holothuriae*

Biology Open • Supplementary information

Table S3. CLC alignment of all identified sequences with database versions.

#Accession_number_Species_and_identifier_Reading_frame_Direction_AA_sequence_of_transcript
>GARW01005949.1_Eucyclops_serrulatus_Eucyclops_serrulatus_contig_5326_1_3_forward
A*VKRTFQYKFTGFQSSSHCQKMPDAVKDGFIRVEDKVYDAEKLADFHPGGPLFVKAFSGRDASQAFLSYHRRQFPHSRAKEA
FHSDDKSVNYKEADNKDFLDLCEIVDKILPRMKSFAPWHFFVKVGLLMAVSLALESYIHVTGSYRWYLTAPLGFLFALIGLNIQHD
ANHGAISRNPKINRAFGATQNWIGGSAISWIHQHVVQHHIHTNDINLDPDIAIEFYVRLNPTHPILKFQIFQYFYFFIIIAFYGLVKV
YMSIEDVITMKHFTPMSTLLQSYQPKELFGVSIFVARWFILPFYFSQGGSWIIAYLNILPIFMTAGYYLSFFFTISHNFRGVHFHEDT
RRESNKQKSFLYNQVVASSNVGGSWLCTLNGGLNYQIEHHLFPRINHTHYPTIAPHVRKFCEDRNIPYVHFPTIGDNLKACIKHL
MDMGNNEQPQTIVFEKTSAKIHLSS*IKGYIYVILTKLDM*VYYVGLQKELNMIVFKL*GVFLTPLYVF*KS*QTIKLTSSISLYFGN
RINLLDSF*SIYLLFKWVLINFEIIYGY*ISKCVFVLITRL*KYCSSFVILLKNYFYISIFRKRFFPKPKILYSRSIPPPPYFINNCQFNLQISY
GII*LKYHFVDSSFFKYHFCQIMLY**ILL*LFFNAIFCIK*NAQILLSFINQCKRYKDIAPRT*Y*VFVNGFRKYCIAPY*VVNKVINRK
K
>GARW01014858.1_Eucyclops_serrulatus_Eucyclops_serrulatus_contig_12979_1_1_reverse
EKLAKMHPGGPMFIHAFSGRDASQAFMSYHRRQFPHSRAAEAFEKVDENVNYTTKDNEDFAELCERVNKVLPRLKSFAPWHY
FLKAFLIMGAAFSLEFYIHYTGSYNWYLTLPLGFIMALIGLNIQHDANHGAISSKPWVNRFFGVTQNWIGGSTVSWIHQHVVQH
HIHTNDVHLDPDIGMEFYVRLNPRHPLLQFHFFQYIYFFIIAALFGIQKVLTSIGDVFSWFHYTPMSMQLKQYAWIEWTATAVYF
TRWIVLPIICSPPGTYFNSMVSVSLMLMVMGWYLSFFFTISHNFSGVQKHEDTTRESNSKKSFLYNQVAASSNVAGFWLAILNG
GLNYQIEHHLFPRINHTHYHTIAPVVREFCEEKQIPYTHFPTVGANVDALIQHLFDLGKNENLNDLLHDPSAKSSARMPLIS*KSK
HLLEPSMLFTIIVSI*KPFSLCLY*SNFSLYVHS*KK*YSYSLLTPFTVCTNYATNKQSLSHQLN*VFFFKNNLDVFHYGFKKE*GGE*
NCLGIRSP*FYLS**CCLLKV*SLN*N*SKILVFRKLAYVYNHFI*T*FSNILISFLFYYNSNKYNSSGKX
>GAZX01027609.1_Caligus_rogercresseyi_tsa-crog-ngs-11111315_2_forward
DNVRT*RLHAQPLENLHRLSK**IVPVVNPSEARNMPPSITKTESIHEASSASFIRSQLPEKPPIGDTSLQVENRIYDAQELAKFHP
GGELFVKAFAGRDATLAFLSYHRKKFPHNRVSFALETVDPTVRDQDVREVDEDYLELCRRVHKVIPSHKTFATWHYYAKVFTLMI
ITLSLEGYMHYYGEYTSLLAISIGLCYAFIGLNIQHDGNHGAISRRPWINRLGGLSMNYIGHSSVDWVHQHVVQHHIYTNDVDLD
PDIDGTFIYRLNPIKPLLQFHALQYIYYFLIINMYGVTLSFYTLENLVKGVHFTSLSTLAQKYWKWESLGPLLLILRLYVLPLIRVPSIW
TLLNMALLMGVFGHYVAFFFILSHNFVGVHHSKGETKIKSFAYKQAACSSNVGGRLLAFFNGGLNYQIEHHLFPRMCHIHYPTV
APIVRAFCEEKSIPYHHFPTITENFVSTAEHLLSLGTEENPESLKFL*RNNNL*YLHKF*IKSRSFVFMFL*S*AIKI*RKFIYKYIL*NSV
S*IS*IIVLNVLQHIYYYYYY*KHSLLTR*AFNVIEKERKKS*GLKIM*CYVVVWHSFLCVCRYISLFLRRKSPDFF*ICCPLAANNEYDE
QANEKEE*DTDT*NNESYFYSIQSC*FISLWTQGEVVFISEAVSYA**NRICFAAAAC*CELDYLLRVSSDVRLMAKFQGSGVRAD
KGVCFHGANARFFVLNMANDSPCL**TVLNF*ALIHD*G*RITNLRFD*VIECVTHVEGYFYFKSLDFRCDNPIGIQGYCFDFDHT
SMPIRYPLRDPQIKCKDFSPFPRNSQSRRACTFQKP*NPPGVLSHFHVKYPFSRVQIS*PNPNIGSHSPRSNQATAFFKFKPHGV
RTSNL*ARGWSSRQDRIS*EDPGGWTYDIRHSGHNMVD**X
>GAZX01029392.1_Caligus_rogercresseyi_tsa-crog-ngs-11113104_3_reverse
RD**IYK*IDRIALQ*RH*VVTLPYVCFCIVQTLLYRP*LYRPCYFFISIKVVSQPFITLKLHKPKLLGLFGSDMLI*FLVMVVAGDEIL
VEGKTYSAQDLAEIHPGGRLFVQAFSGLDASVAFLSYHRRKFPHSRAKTALKAEGNDTNNPEGWDDEYLELCRLVEKVLPRSKSF
APWYYYLKVAVILSSALSIELFMHFHASYPPLLCAFLGYIFAVIGLNIQHDANHGAVSRRPWVNRMLGMSQNWIGGSAINWIH
QHVVQHHIHTNDIDQDPDMDGGILIRINPKTPLMKFHIVQHIYFFFLLAFYGFSVVIRSLLNVIYGEHFTPMSVLLKAHRVFEVFM
SLFFISRLLLFPLYQSPNLRTLGSLLLMFMVAGYYLSLFFAISHNFEGVELNHEREKPHSFLRNQVATSSNVGGEILCFLNGGLNYQI
EHHLFPRISHCHYPKIAPVVKEFCQARNIPYVHFPSVLDNVNSSIRHLIFMGGASEPFPKLSAQRLATTKLQS*RTPH*L*A*VFICR
DILTSSHLK*IKPLYKICHLCVINRDIQK**DSPDRLFSYPAKTKEKTCENNGYI**EAPPTLHLFFFCFNGFQIMLIT*KMINFPALFK
NFEQRLA**FFKFQLKKLPPY*TRARKEERGFHRFRVLFIGSSL*CKSCSPWAFKTSQKTTLMMPNRPLTSLV

Biology Open • Supplementary information

>GAZX01029393.1_Caligus_rogercresseyi_tsa-crog-ngs-11113105_3_reverse
SSPHNWLLPTCFSSSM*LLSYD*AIPFFDYMSAFFLLLDMSV*FLVMTVAGDEILVEGKTYSAQDLAEMHPGGRLFVQAFSGLD
ASAAFLSYHRRKFPHSRAKTALKAEGNDTNNPEGWDDEYLELCRLVEKVLPRSKSFAPWYYYLKVAVILSSALSIELFMHFHASYP
PLLCAFLGYIFAVIGLNIQHDANHGAVSRRPWVNRMLGMSQNWIGGSAINWIHQHVVQHHIHTNDIDQDPDMDGGILIRINP
KTPLMKFHIVQHIYFFFLLAFYGFSVVIRSLLNVIYGEHFTPMSVLLKAHRVFEVFMSLFFISRLLLFPLYQSPNLRTLGSLLLMFMV
AGYYLSLFFAISHNFEGVELNHEREKPHSFLRNQVATSSNVGGEILCFLNGGLNYQIEHHLFPRISHCHYPKIAPVVKEFCQARNIP
YVHFPSVLDNVNSSIRHLIFMGGASEPFPKLSAQRLATTKLQS*RTPH*L*A*VFICRDILTSSHLK*IKPLYKICHLCVINRDIQK**
DSPDRLFSYPAKTKEKTCENNGYI**EAPPTLHLFFFCFNGFQIMLIT*KMINFPALFKNFEQRLA**FFKFQLKKLPPY*TRARKEE
RGFHRFRVLFIGSSL*CKSCSPWAFKTSQKTTLMMPNRPLTSLV
>GBFB01149783.1_Calanus_finmarchicus_comp270289_c0_seq6_1_reverse
QAFLTYHRRQFPHTSDIAKAAFEEVDDTVKYTKDDNQDFMELCDRVAKVLPRLKSFAPWHYYLKVAFLLSFVIGLESYMHYNAV
YRWELSAIMGLFYAWIGLNIQHDANHGAVSRYPGVNRVLGMSQNWIGGSALSWVHQHVVQHHLHTNDVNLDPDMAGSSY
IRLNPLKPLMGYHIVQHIYFFGLLAVYGFSVVIQSFLQVLYGAHHTAMSPLLVPHRIFESFMSVFFICRWVFLPLYQVPQLSTIWST
LPMYMVAGYYLAFFFSISHNFKDVYMMEDTTREASNHKNGTFLYKQVVSSSNVGGPILCFFNGGLNYQIEHHLFPRISHCHYPVI
APIVREYCKEKSIPYVHFDTIAENIRSCALHLLDLGSHQTPKNVHFIQKEKSQ*INTIYIKKTX
>GBSZ01004544.1_Tigriopus_californicus_SD_As4_Contig22385_1_forward
MSATKLAADQILVEGKVYSSTKLADLHPGGPLFLRAFSGRDATQAFLSYHRRTFPHSRVKEAFEHHHPDVVYDPATNADYLELCE
RIDKVIPRMKSFAPWSYFIKATLIWSTAIGLEIHMHYNARYPWFETALLGFFFALMGLNVQHDANHGSLSRKPWVNRFWGLAQ
NWYGGSSVSWIHQHVVQHHIHTNDVFLDPDIEGKSPILRLNPNQPLLKAYAFQHVHYFLLILVFGYNIIYQAVTTVLEFANKAPF
SVLLRPYILGEHIWNVFFVVRWFLLPMVLAPSLWTPLTIMPMFMVFGTYLSFFFHISHNFDGVEQLEDTSSKNSFLYNQLITSSN
VCGAKLCFLNGGLNYQIEHHLFPRMHHSHYPTVAPHVRNFCEEKGLPYHHFPTIRENLSSCINHLSDFGRK
>GBSZ01004578.1_Tigriopus_californicus_SD_As4_Contig8388_1_forward
LADLHPGGPLFIKAFSGRDASQAFLTYHRRQFPHQRVKEAFESTDETVTYSTDDHADFIDLCERIEKVLPRNKSFAPWHYYVKVA
FIMGSVFSLEAYMHYTKSYNWQLFAVMGWFYALIGLNIQHDANHGAISRNPVINRVLGASQNWIGGSAIAWIHQHVVQHHI
HTNDVHLDPDIAGSIYIRLNPLKPLLKYHVVQHIYFFFLLALYGFSIVIQSLENVVSGAHHTPMSPLLKPHRVFETFMWSLFFLRWV
ITPVYQTGTFLTLLHSLPMYMVGGYYLAFFFSISHNFKGVHILEDTTRPSNKESSFLYKQVVSSSNVGGPFLCFMNGGLNYQIEHH
LFPRISHTHYPKIAPTVKAFCLEKNIPYVHFPTIGENIRSCTQHLWDMGSNETPKNATIQAAKTLLVN
>GBTC01004500.1_Tigriopus_californicus_SCN_Contig_30034_1_forward
MSAPKLAADQILVEGKVYSSTKLADLHPGGPLFLRAFSGRDATQAFLSYHRRTFPHSRVKEAFEHHHPDVVYDPATNADYLELCE
RIDKVIPRMKSFAPWSYFIKATLIWSTAIGLEIHMHYNASYPWFETALLGFFFALMGLNVQHDANHGSLSRKPWVNRFWGLAQ
NWYGGSSVSWIHQHVVQHHIHTNDVFLDPDIEGKSPILRLNPNQPLLKAYAFQHVHYFLLILVFGYNIIYQAVTTVLEFANKAPF
SVLLRPYILGEHIWNVFFVVRWFLLPIVLAPSLWTPLTIMPMFMVFGTYLSFFFHISHNFDGVEQLEDTSSKNSFLYNQLITSSNVC
GAKLCFLNGGLNYQIEHHLFPRMHHSHYPTVAPHVRNFCEEKGLPYHHFPTIRENLSSCINHLSDFGRK
>GBTC01004534.1_Tigriopus_californicus_SCN_Contig_11004_1_forward
LADLHPGGPLFIKAFSGRDASQAFLTYHRRQFPHQRVKEAFESTDETVTYSTDDHADFIDLCERIEKILPRSKSFAPWHYYVKVAFI
MGSVFSLEAYMHYTKSYNWQLFAVMGWFYALIGLNIQHDANHGAISRNPVINRVLGASQNWIGGSAIAWIHQHVVQHHIHT
NDVHLDPDIAGSIYIRLNPLKPLLKYHVVQHIYFFFLLALYGFSIVIQSLENVVSGAHHTPMSPLLKPHRVFETFMWSLFFLRWVIT
PVYQTGTFLTLLHSLPMYMVGGYYLAFFFSISHNFKGVHILEDTTRPSNKESSFLYKQVVSSSNVGGSFLCFMNGGLNYQIEHHLF
PRISHTHYPKIAPTVKAFCLEKNIPYVHFPTIGENIRSCTQHLWDMGSNETPKNATIQAAKTLLVN
>GCHA01001884.1_Tigriopus_japonicus_TJ_CDS_01884_1_forward

ACFVLTFCWRTDLSFLGLLLSDKATCEGFSVTSYRILEMAPNATSQDALVAETAEDCFSGGHLPIGDESIRVEDKIYSAKTLANMH
PGGPLFIKSFAGRDASQAFLSYHRRQFPHSRVKEALEKVDPNVKVDPHAQDDFLELCERVDKVLPRMQSFAPWSYYLKASFIMG
MAFSLEFYMHYTGNYRWYISGLCGWFFALIGLNIQHDANHGALSRRSWVNRLWGLSQNWIGGSTISWIHQHVVQHHIHTND
IKLDPDIEGRVVVRLNPRKPVLKWHFLQHIYFFFIILGFGFSVILSSFTTMLDGINYTPVSKMLNRQRYLEMSWSILHFIRWIVVPLY
QAPSIYTFFNCAPMYIVVGWYLSFFFTLSHNFDGVTQLDNTQPSKSLLYNQVITSSNVGGKFLCFLNGGLNYQIEHHLFPRMHH
GHYPKIAPVVKQFCAEKGIPYQHFPTVSSNAWACAKHLLEMGTQDVPKSAKHLVTS*
>GCHA01019274.1_Tigriopus_japonicus_TJ_CDS_19276_1_forward
MAPNSEIHPESIRVEDRIYSAKKLAEFHPGGPLFIKAFSGRDASQAFLTYHRRQFPHQRAQEAFERVDQTVTYSTDDHADFIELCE
RIEKVLPRNKSFAPWHYYVKVAFIMGTVFCLEAYMHMTKSYNWQLFAVMGWFYALIGLNIQHDANHGAISRNPVINRVLGAS
QNWIGGSAIAWIHQHVVQHHIHTNDVHLDPDISGSMYIRLNPLKPLLKYHVVQHIYFFFLLALYGFSVVVQSLENVVSGAHHTP
MSPLLKPHRVFETFMWSLFFLRWVITPVYQTGTFLTLLNTLPMYMVGGYYLAFFFSISHNFKGVHILEDTTRPSNKESSFLYKQVV
SSSNVGGSFLCFMNGGLNYQIEHHLFPRINHTHYPKIAPTVKAFCLEKKIPYVHFPTIGENIRSCTQHLWDMGSHETPKNATIQA
AKAMLAH*
>GCHA01019336.1_Tigriopus_japonicus_TJ_CDS_19338_1_forward
MAPNSEIHPESIRVEDRIYSAKKLAEFHPGGPLFIKAFSGRDASQAFLTYHRRQFPHQRAQEAFERVDQTVTYSTDDHADFIELCE
RIEKVLPRNKSFAPWHYYVKVAFIMGTVFCLEAYMHMTKSYNWQLFAVMGWFYALIGLNIQHDANHGAISRNPVINRVLGAS
QNWIGGSAIAWIHQHVVQHHIHTNDVHLDPDISGSMYIRLNPLKPLLKYHVVQHIYFFFLLALYGFSVVVQSLENVVSGAHHTP
MSPLLKPHRVFETFMWSLFFLRWVITPVYQTGTFLTLLNTLPMYMVGGYYLAFFFSISHNFKGVHILEDTTRPSNKESSFLYKQVV
SSSNVGGSFLCFMNGGLNYQIEHHLFPRINHTHYPKIAPTVKAFCLEKKIPYVHFPTIGENIRSCTQHLWDMGSHETPKNATIQA
AKAMLAH*
>GCHA01047837.1_Tigriopus_japonicus_TJ_CDS_47840_1_forward
MAPNATSQDALVAETAEDCFSGGHLPIGDESIRVEDKIYSAKTLANMHPGGPLFIKSFAGRDASQAFLSYHRRQFPHSRVKEALE
KVDPNVKVDPHAQDDFLELCERVDKVLPRMQSFAPWSYYLKASFIMGMAFSLEFYMHYTGNYRWYISGLCGWFFALIGLNIQ
HDANHGALSRRSWVNRLWGLSQNWIGGSTISWIHQHVVQHHIHTNDIKLDPDIEGRVVVRLNPRKPVLKWHFLQHIYFFFIIL
GFGFSVILSSFTTMLDGINYTPVSKMLNRQRYLEMSWSILHFIRWIVVPLYQAPSIYTFFNCAPMYIVVGWYLSFFFTLSHNFDGV
TQLDNTQPSKSLLYNQVITSSNVGGKFLCFLNGGLNYQIEHHLFPRMHHGHYPKIAPVVKQFCAEKGIPYQHFPTVSSNAWACA
KHLLEMGTQDVPKSAKHLVTS*
>GCHA01047861.1_Tigriopus_japonicus_TJ_CDS_47864_1_forward
MPDVGKVSDTVEKLPWDHIRIGNKSYDAKKLSGFHPGGPLFISAFAGRDASQAFVSYHRKPFPHQKVKFALEKEDPNIPLPDPH
LNDDFFELVQRVEKVLPRQKSFATWSFYLKAMILLTICFGAEAYMHYTVTYKWYLTALLGMMEAIMALNITHDALHGSLSANPR
VNRFFSLFHHVYSGTVTTWVHQHVVQHHVHTNDVHRDPDISGQRAMRLNPLRPLKSMHSYQYIYFFALFFIYGLTIFNFTISTV
AKAYYFTAFSPMLQKYRIFDAIGLAIFHFRWTILPVYQTQSLFTLFHTVPLMIGFGTYMATFFHISHNFEGVEQFEDSGPGKSWLY
HQVASSSNVAGSILCTINGGLNYQIEHHLFPRVYHGHYPTIAPIVRQFCEERSIPYRHFPTLTDNMASTVKHMYDFGVNKVPDAA
KNLKGLGTQSAMGVPLSG*
>GCHA01047992.1_Tigriopus_japonicus_TJ_CDS_47995_1_forward
MSGSESNMTKERRVGSDFVASSLTREKVDVMMDCPANQVRIGNKFYNAEKLLDFHPGGQLFISAFAGRDASQAFMSYHRKRF
PHQKMKFALDNEDTLAPKEDPHLNDDYFDLVERIEKVLPRHKSFAPWTYYLKAFFIIGVCLALEGYMHYTGHYDWHLSAILGLFY
AYLGLNIQHDANHGALSANPLVNRLMGMTQNWYGASVTSWVHQHVVQHHVHTNDVHLDPDIEGKVVMRLNPMRPLKKL
HAYQWFYFFGLIFLYGFSIVKFSFTTVLEGFYYTPFSELLNRYRYQELAWSLFFYFRWFFLPYYMAPTWTTFFQIAPMFMIFGFYLS

Biology Open • Supplementary information

VFFHISHNYLGVEQLEDTSPKRSWLHNQVVASSNVAGPILGFLNGGLNYQIEHHLFPRIHHSHYPTIAPVVRQFCEEKGIPYRHFE
TVTENVISSIQHMYDIGTNVVPEAAKKLENVKMHARTLSVAG*
>GCJT01017122.1_Paracyclopina_nana_Pnana_mRNA_18686_1_forward
MEPDHGVMDKKHIRVEGKIYSAEKLAELHPGGPLFIQAFSGRDASQAFLSYHRRQFPHKRAEPAYISDDTTVSHDPQDHADFLE
LCQRVDKVLPRMKSFAPWHYYIKVAFILGSAFGLELYMHINRAYVWYLSALVGLFYALIGLNIQHDANHGAISRNPWVNRILGM
SQNWIGGSSISWIHQHVVQHHIHTNDLERDPDIAGNAYIRLNPNQKLMRFHIVQHVYFFFLMAIYGFSVVIQTVDNILKGKHHT
TMSVLLGPHRAFEAVTSALFILRWMVLPVYLTGSFMTLLHTVPMYIVAGYYLAFFFTISHNFEGVHMMEDTRRGFNSKSSFLYN
QVVTSSNVGGAFLCMLNGGLNYQIEHHLFPRIQHSHYPKIAPVIRAFCEEKGIPYVHFDSINENMASCVKHLIDLGNNVNPVHIT
MISAAN*
>GDFW01009453.1_Tigriopus_sp._1_SL-2012_Tk_CDS_08242-1_1_forward
LANGLVIFISFHQRSPEGPEVLKMCKPDVKKDLNPTEDEIQIGNKFYDVDKLKKFHPGGPLFISAFAGRDATQAFISYHRKQFPHQ
KVEFALERTEKAVEDYDPSVNEDYFELIRRVEKVVPRHKSFATWAYHFKAFVMVSIVVALEAYIHYTGTYKWYLTGALGIGLAIAA
LNLIHDAHHGALSKNPKVNRLYGSLQHWYGHCVTTWIHQHVVQHHIHTNDIHKDPDINEQKAVRLNPVYPMMKFHAYQWA
YFFGLFFLFGFSMIAFSLKTALNGFHFTPFSKHLKEYRTLDLVGYWIYYTRWLVIPLIRFPSIWTFLNILPLFITFGFYLVFFFYISHNFA
GVTLLDDTSPNQSWLYNQVTTSSNVAGPILCFINGGLNYQIEHHLFPRMYHGHYPTIAPVVREFCKEKGIPYRHFPTLTSNFIAT
MEHMYDFGNNVVPESTKNLEIKKRS*
>GDFW01009455.1_Tigriopus_sp._1_SL-2012_Tk_CDS_08243-1_1_forward
MASDLSPESIRVEDKIYSARLLAELHPGGPLFVQAFAGRDASTAFLTYHRRQFPHQRAAPAFQSKDESVQYSPDDHADFIELCER
VDKVLPRMKSFAPWHYFVKVGVILGAAFTLEAYMHLTQSYRWFLFAIMGLLYALIGLNIQHDANHGAISRNPWVNRVLGTSQ
NWIGGSAIAWIHQHVVQHHIHTNDIHLDPDISGSTYIRLNPLKPLLKYHFVQHIYFFILLALYGFSIVIQSLENVVSGSHHTPMSPLL
KPHRVFETFTWVLFITRWVVLPVAQTGSLSVLLQTLPLYMVAGYYLAFFFSISHNFNGVHLLEDTTRPSNKDSSFLYKQVVSSSNV
GGSILCFMNGGLNYQIEHHLFPRISHTYYPKIAPTVREFCKEKNIPYVHFPTLGENIRSCTQHLWDMGSHETPKNATIQAAKTML
AH*
>GDFW01015540.1_Tigriopus_sp._1_SL-2012_Tk_CDS_13549-1_1_forward
MAPNATGSELRGDGSQECFPGDPLPIGEESIRVEDKIYSARTLADLHPGGTLFVKSFAGRDASTAFLSYHRRQFPHSRVKEALEKV
DPNVKVDPHAQDDFLELCERVDKVLPRMKSFAPWTYYLKACFIMFMTVGLEFYMHYTGNYKWYISALCGWFCALVGLNIQHD
ANHGALSRNPLVNRFWGLSQNWIGGSTISWIHQHVVQHHIHTNDVKLDPDIEGRPVVRLNPRKPVLKWHFLQHIYFFILILGYG
FSVILSSFTTMLEGINYTPVSKLLKNHRYLEMAFSILYFFRWVVIPLYQVPSLFTILHCAPVLMVTGWYLSFFFTLSHNFDGVTQLD
NTQPSKSLLYNQVITSSNVAGEWLCFLNGGLNYQIEHHLFPRMHHGHYPKIAPVVREFCQEKGIPYHHFPTITENAWACAKHLF
EMGTEDVPKSAKHIMAS*
>GFUD01041922.1_Neocalanus_flemingeri_Contig41928_2_forward
CSTQPDLLPVCVL*GE*LV*PELVIVIPVIGERGVIL*ER*DIVSCRVSADTQHPEGVSDMSPKSNEMMDNHLLQSESLTRPGPVA
AEYIRVESKIYDARRLAELHPGGPLFIKAFAGRDASQAFLSYHRRQFPHSSTKAKAAYERDDETVKYSKEDNQDFMELCDRVEKV
LPRLSSFAPWYYYLKVAFLLCFVVTLEGYMHYNRVYSWKLSALMGLFYAWIGLNIQHDANHGAVSRYHWVNRALGASQNWIG
GSAISWVHQHVVQHHMHTNDVNLDPDMAGSAFIRLNPLAPLMKYHIVQHIYFFFLLAIYGFSVVIQSFLNVVYGSHHTAMSPL
LVPHRVFESFMSIIFVCRWVLLPLYQVPELDTFLSTVPMYMVCGYYLAFFFSISHNFSGVFMLEDTTREASSEDTGTFLYKQVVSS
SNVGGRVLCFFNGGLNYQIEHHLFPRISHCHYPTISHTVHQYCQEKGIPYVHFNTVWENIRSCTKHLLDLGSHQTPKNVNFFEQK
VKSQ*ARK*ELK*MKMLCNGLR
>GFUD01115445.1_Neocalanus_flemingeri_W4a28933:c0_g1_i2_2_forward

Biology Open • Supplementary information

VTSYTYSEEREWGEES**SLLSLSMGREELYWY*HSSS*RVSDMSPKSNEMMDNHLLQSESLTRPGPVAAESIRVESKIYDARRL
AELHPGGPLFIKAFAGRDASQAFLSYHRRQFPHSSTKAKAAYERDDETVKYSKEDNQDFMELCDRVEKVLPRLSSFAPWYYYLK
VAFLLSFVVTLEGYMHYNRVYSWKLSAIMGLFYAWIGLNIQHDANHGAVSRYHWVNRALGASQNWIGGSAISWVHQHVVQ
HHMHTNDVNLDPDMAGSAFIRLNPLKPLMKYHIVQHIYFFFLLAIYGFSVVIQSFLNVVYGSHHTAMSPLLVPHRVFESFMSIVF
VCRWVLLPLYQVPEIDTFLSTVPMYMVCGYYLAFFFSISHNFSGVFMLEDTTREASSADTGTFLYKQVVSSSNVGGRVLCFFNGG
LNYQIEHHLFPRISHCHYPTIAHTVHQYCQEKGIPYVHFNTVWENIRSCTKHLLDLGSHQTPENVSFFEQKVKSQ*ARK*ELDCM
KMLHNGLRI*FNNFVFNS*IINFSVKCSQITPN*AKTSSPPQ
>HACA01025139.1_Lepeophtheirus_salmonis_mRNA_for_putative_protein,_similar_to_XP_004994750.1,_contig_Lsa.22072,_3_forward
KYSAFL*KIKAVSI*SLV*QVVMAPSITTTETIETLKVDTKSKTKPIIGQNSLQVENKIYDAEELARIHPGGKLFVKAFAGRDATTAFL
SYHRKKFPHNKVSFALEGIDPTVSEKDIRDVDEDYLELCRRVHRVIPRHKTFAPWHYFVKIFALMIITLFLEYNMHYTGAYTWYYA
SSIGLCYALIGLNIQHDGNHGAVSSKPWLNRLAGLSMNFIGHSSVDWVHQHDVQHHIYTNDVDLDPDIDGTPLYRLNPLKPLLK
IHGLQYIYYFMIINLYGVTVSFFTLSNVIQGMHFTSLSTLAQKYWKLEALGPMLLILRMYIIPIIRVPSIWTVLNIFLLMGTFGQYVA
FFFILSHNYVGVHHTQGETKTKSFIYKQVACSSNVGGKLLCFFNGGLNYQIEHHLFPRMCHVHYPTVAPIVRQFCKEKSIPYNHFP
TITENFVSTAEHLLGLGTEIKPTTMKFL*I*IWPALNY*LYLFMTIVGCPPHMFLFL*RSNIIIAKMX
>HACA01032486.1_Lepeophtheirus_salmonis_mRNA_for_putative_protein,_similar_to_XP_004994750.1,_contig_Lsa.28773_1,_3_forward
FSRKTSCKLDF*IYKRFCWFMANDI*CGF*YHSTPPFMTSV**D*LEKRDILIF*RVIMTPKVAKDEILVEGKIYSAENLAQSHPGG
KLFVRAFAGLDASTAFLTYHRRKFPHSRAKNALKEDGEDSNNPEGWDEEYLELCRLVEKVIPRMKSFAPWYYYVKVAIILSMAVS
IEYYMHKNVFYHPLLCGFLGYIFAVIGLNIQHDANHGAISRRPLVNRILGMSQNWIGGSAINWIHQHVVQHHIHTNDIDQDPD
MDGGILIRINAKTPLMKFHIVQHIYFFFLLSFYGFSVVIRSLLNVIVGKHFTPMSSLFKKYRVIEVFSSVFFILRLMVFPIYQAPEIQTV
LSLSLMFIVAGYYLSLFFAISHNFEGVEMNMKNAKSSSILRTQVATSSNVGGEILCFLNGGLNYQIEHHLFPRISHCHYPKIAPVVR
QFCEERNIPYVHFPSVLDNINSAIKHLVFMGGNAEPFPELNTHILASKLQS*SVIIILLFSLDYKSLKSIIPLIHIYHLL*YGSSPPPSX
>HACA01032487.1_Lepeophtheirus_salmonis_mRNA_for_putative_protein,_similar_to_XP_004994750.1,_contig_Lsa.28773_2,_1_forward
IKTTLYFQSFF*GKLLVLNECLYITQCCFLIMDILIF*RVIMTPKVAKDEILVEGKIYSAENLAQSHPGGKLFVRAFAGLDASTAFLTY
HRRKFPHSRAKNALKEDGEDSNNPEGWDEEYLELCRLVEKVIPRMKSFAPWYYYVKVAIILSMAVSIEYYMHKNVFYHPLLCGFL
GYIFAVIGLNIQHDANHGAISRRPLVNRILGMSQNWIGGSAINWIHQHVVQHHIHTNDIDQDPDMDGGILIRINAKTPLMKFHI
VQHIYFFFLLSFYGFSVVIRSLLNVIVGKHFTPMSSLFKKYRVIEVFSSVFFILRLMVFPIYQAPEIQTVLSLSLMFIVAGYYLSLFFAIS
HNFEGVEMNMKNAKSSSILRTQVATSSNVGGEILCFLNGGLNYQIEHHLFPRISHCHYPKIAPVVRQFCEERNIPYVHFPSVLDNI
NSAIKHLVFMGGNAEPFPELNTHILASKLQS*SVIIILLFSLDYKSLKSIIPLIHIYHLL*YGSSPPPSX
>HAHV01077442.1_Tisbe_holothuriae,_strain_RUC.THI1,_contig_TRINITY_DN61994_c0_g1_i1,_3_reverse
KSIGPPFLVPAKTESSTTKNVRKYLKPGDSLEDNSFPNQKSICESVQGGSMCPTPKSKSADKLVEAVGRTGGKENSRVTGHDEDN
FGLKTTWQQTAEVDPEQILIEDKFYDAKGLANFHPGGPLFLKAFAGRDATQAFLSYHRKKFPHQRVEKYLDHQDSSIEYDGNED
FLELCKRVEKVIPRSKSFAPWSYYLKAIFIWGTAIWLEARMHQNGNYPWYEAVILGFFWALIGLNIQHDANHGSLSANPVVNRF
WGLGNNWYGHSAISWIHQHVVQHHVHTNDVHLDPDIEGKSPILRLNPYQPLETSYLYQHIYYFLLILAFGYNIIYQAVLTIITYTN
KSIFSSLLKPYILQEHLWNIFFIFRWFLLPLFTSGGDPWVYARIFPMFAVFGSYLSFFFHISHNFQGVEQLEDTSAKHSFLYNQLITSS
NVCGWKLAVLNGGLNYQIEHHLFPRMHHSHYATASPIVREFCREKGYPYHEFKTIAENVRSCIKHLRDYGSNRIPEAAQKLNIAEI
KKEQNEX
>GHAJ01038079.1_Apocyclops_royi_TRINITY_DN52334_c0_g1_i1_2_[moltype=transcribed_RNA]_2_forward

Biology Open • Supplementary information

NRFGSQTKQILLRS*KKVVSETF*PVSKNNTAQTLNFVYKCTRRKHR*HRNSSFTFILSTI*IFHLTMPSRVPDELIRVEDKLYSAQQ
LAKIHPGGPLFVKAFAGRDSSQAFLSYHRKPFPHNRAKEAFYAVDKSVDYSEKDNQDFLELCELINKVLPKGKSFAPWHYFVKIGI
LTLVTVILEFYIHYTASYKWHLTALLGFLFALIGLNIQHDANHGAISLNPNVNRFLGTFQNWIGGSAISWIHQHVVQHHIHTNDV
RLDPDIALEFYVRLNPTHPLLKFQLFQYFYFFILIAFYGVLKVVLSIEDVLSWKHYTPMS
>GHAJ01038078.1_Apocyclops_royi_TRINITY_DN52334_c0_g1_i2_1_[moltype=transcribed_RNA]_1_forward
MKLSLEDVEN*VPQNLSSVTKQIFHLTMPSRVPDELIRVEDKLYSAQQLAKIHPGGPLFVKAFAGRDSSQAFLSYHRKPFPHNRA
KEAFYAVDKSVDYSEKDNQDFLELCELINKVLPKGKSFAPWHYFLKIGILTLVTVILEFYIHYTASYKWHLTALLGFLFALIGLNIQHD
ANHGAISLNPNVNRFLGTFQNWIGGSAISWIHQHVVQHHIHTNDVRLDPDIALEFYVRLNPTHPLLKFQLFQYFYFFILIAFYGVL
KVVLSIEDVLSWKHYTPMS
>GHAJ01038077.1_Apocyclops_royi_TRINITY_DN52334_c0_g2_i2_1_[moltype=transcribed_RNA]_1_forward
IIYTPDGRWWISLVNILPMAMVAGYYLSFFFTISHNFRGVHMHEDTRRETNQKNSFLYNQVVSSSNVGGSWLCFLNGGLNYQI
EHHLFPRINHTHYPTIAPHVKKFCDERKIPYVHFTTIDANLRACIKHLMDMGESEMPNSVVMEKAATKMPIVS*AT*CPSTLQH
PTPPDVQSPPPENEKTY**KKKDFLRLHCYVAK*HTIRVQKQTQLPEKNSSMCTYIFNYSEEVAV*KIVHLSCLKSNITF*MDPAH
CHCPKALLFRWGSIEAYFI*ATLF*ELRRQPIAAIGISMTLAGLSAESTILNMTATVWE*RQGSEFCASIKKLCEIMDDVRWKFFIM
ND*SIINYYNQKYVLINYKESSAA*EDSLEFSKKILLRETKSEIFNYF*STSIIS*LLNRIEGY*LKWQVLRS*LQFSF*N*FFLLCLISTIY
CSNPMQCKTIKCFFYCVFIRVLGIRNMERLKVQPWYDRRVR*SSTIVPNNFVPRIWSSILLYFAAVLSYHTGKKGPP
>GHAJ01039406.1_Apocyclops_royi_TRINITY_DN55045_c0_g3_i1_3_[moltype=transcribed_RNA]_3_forward
IIILGAAFSLEFYIHFTGSYIWYLTAVLGFVFALIGLNIQHDANHGAISRRAWVNRLFGKSQNWIGGSTVSWIHQHVVQHHVHTN
DLELDPDIAIEFYIRLNPRHPLLKFHVFQYIYFFLIAALFGLQKIMTSLGDVLSWHHYTPIANELRKYAWEEWIFTVVYIIRWVVLPFF
MVAPGQAVNYVFSWVVMNMVMGYYLSFFFTISHNFEG
>GHAJ01039408.1_Apocyclops_royi_TRINITY_DN55045_c0_g3_i2_1_[moltype=transcribed_RNA]_1_forward
YTTKDNDDFLDLCSRVEKILPRSKAFAPWHYYLKAIIILGAAFSLEFYIHYTGSYIWYLTAVLGFVFALIGLNIQHDANHGAISRRAW
VNRLFGKSQNWIGGSTVSWIHQHVVQHHVHTNDLELDPDIAIEFYVRLNPRHPLLKFHVFQYIYFFLIAALFGLQKIMTSLGDVL
SWHHFTPIANELRKYAWEEWIFTVVYIIRWVVLPVFMVSPGQAVNYVFSWVVMNMVMGYYLSFFFTISHNFEGVEX
>GHAJ01039407.1_Apocyclops_royi_TRINITY_DN55045_c0_g5_i1_3__[moltype=transcribed_RNA]_3_forward
HLFPRINHTHYYYIAPVVRQFCEERKIPYTHFESVPQNVRALVQHLAEMGSNKNFNIEGFVNPSEKVSAKMHIVS*TPKILWFVEL
*NRQKASFDVATKTESITRYNFRISIPCIEMKISPC*QIKCILLF*QSSNINAEIYVF*SITHFFYINV*LT*TY*RDAVNDLILIIKS*LGT
ILK*EIVLRSSKKLRRYSYFTHFSKNFSPSLKVVLAA
>NA_Apocyclops_royi_NODE_1_length_2498_cov_171.314227_g0_i0_complete_gene_3_reverse
ETF*PVSKNNTAQTLNFVYKCTRRKHR*HRNSSFTFILSTI*IFHLTMPSRVPDELIRVEDKLYSAQQLAKIHPGGPLFVKAFAGRD
SSQAFLSYHRKPFPHNRAKEAFYAVDKSVDYSEKDNQDFLELCELINKVLPKGKSFAPWHYFLKIGILTLVTVILEFYIHYTASYKW
HLTALLGFLFALIGLNIQHDANHGAISLNPNVNRFLGTFQNWIGGSAISWIHQHVVQHHIHTNDVRLDPDIALEFYVRLNPTHPL
LKFQLFQYFYFFILIAFYGVLKVVLSIEDVLSWKHYTPMSRLLKSYQSFEVLGSAVFMLRWIALPIIYTPDGRWWISLVNILPMAMV
AGYYLSFFFTISHNFRGVHMHEDTRRETNQKNSFLYNQVVSSSNVGGSWLCFLNGGLNYQIEHHLFPRINHTHYPTIAPHVKKF
CDERKIPYVHFTTIDANLRACIKHLMDMGESEMPNSVVMEKAATKMPIVS*AT*CPSTLQHPTPPDVQSPPPENEKTY**KKKD
FLRLHCYVAK*HTIRVQKQTQLPEKNSSMCTYIFNYSEEVAF*KIVHLSCLKSNITF*MDPAHCHCPNALLFRWCSIEAYFI*ATLF*
ELRRQPIAAIGISMTLAGLSAESTILNMTATVWE*RQGSEFCASLKKLCEIMDDVRWKFFIMND*SIINYYNQKYVLINYKESSAA
*EDSLEFSKKILLRETKSEIFNYF*STSIIS*LLNRIEGY*LKWQVLRS*LQFSF*N*FFLLCLISTIYCSNPMQCKTIKCFFYCVFIRVLGI
RNMERLKVQPWYDRRVR*SSTIVPNNFVPRIWSSILLYFAAVLSYHTGKKGP
>NA_Apocyclops_royi_NODE_1_length_1925_cov_185.031857_g0_i0_complete_gene_1_reverse

Biology Open • Supplementary information

FLLQMIELFVFFKVF*TVLADRAIFALPLSEKICLPQSSKSENQNNSFLGVQGFRIRKDCINMSEVPEGQIRIEDKVYSAEKLSKLHP
GGPVFIKAFSGRDASQAFLSYHRRNFPHGSKPAKESFLLQDKSIKYTTKDNDDFLDLCSRVEKILPRSKAFAPWHYYLKAIIILGAAF
SLEFYIHFTGSYIWYLTAVLGFVFALIGLNIQHDANHGAISRRAWVNRLFGKSQNWIGGSTVSWIHQHVVQHHVHTNDLELDP
DIAIEFYIRLNPRHPLLKFHVFQYIYFFLIAALFGLQKIMTSLGDVLSWHHYTPIANELRKYAWEEWIFTVVYIIRWVVLPFFMVAP
GQAVNYVFSWVVMNMVMGYYLSFFFTISHNFEGVEKHEDTRRPVNAHKSFLYNQVAASSNVAGFWLAILNGGLNYQIEHHL
FPRINHTHYYYIAPVVRQFCEERKIPYTHFESVPQNVRALVQHLAEMGSNKNFNIEGFVNPSEKVSAKMHIVS*TPKILWFVEL*
NRQKASFDVATKTESITRYKFRISIPCIEMKISPC*QIKCILLF*QSSNINAEIYVF*SITHFFYINV*LT*TY*RDAVNDLILIIKS*LGTIL
K*EIVLRSSKKLRRYSYFTHFSKNFSPSLKVVLAA
>NA_Apocyclops_royi_NODE_2_length_1925_cov_171.963823_g0_i1_complete_gene_1_reverse
FLLQMIELFVFFKVF*TVLADRAIFALPLSEKICLPQSSKSENQNNSFLGVQGFRIRKDCINMSEVPEGQIRIEDKVYSAEKLSKLHP
GGPVFIKAFSGRDASQAFLSYHRRNFPHGSKPAKESFLLQDKSIKYTTKDNDDFLDLCSRVEKILPRSKAFAPWHYYLKAIIILGAAF
SLEFYIHYTGSYIWYLTAVLGFVFALIGLNIQHDANHGAISRRAWVNRLFGKSQNWIGGSTVSWIHQHVVQHHVHTNDLELDP
DIAIEFYVRLNPRHPLLKFHVFQYIYFFLIAALFGLQKIMTSLGDVLSWHHFTPIANELRKYAWEEWIFTVVYIIRWVVLPVFMVSP
GQAVNYVFSWVVMNMVMGYYLSFFFTISHNFEGVEKHEDTRRPVNAHKSFLYNQVAASSNVAGFWLAILNGGLNYQIEHHL
FPRINHTHYYYIAPVVRQFCEERKIPYTHFESVPQNVRALVQHLAEMGSNKNFNIEGFVNPSEKVSAKMHIVS*TPKILWFVEL*
NRQKASFDVATKTESITRYKFRISIPCIEMKISPC*QIKCILLF*QSSNINAEIYVF*SITHFFYINV*LT*TY*RDAVNDLILIIKS*LGTIL
K*EIVLRSSKKLRRYSYFTHFSKNFSPSLKVVLAA

Biology Open • Supplementary information
